# Glutathione levels are associated with methotrexate resistance in acute lymphoblastic leukemia cell lines

**DOI:** 10.3389/fonc.2022.1032336

**Published:** 2022-12-01

**Authors:** Rafael Renatino Canevarolo, Carolina Pereira de Souza Melo, Nathalia Moreno Cury, Leonardo Luiz Artico, Juliana Ronchi Corrêa, Yanca Tonhasca Lau, Samara Sousa Mariano, Praneeth Reddy Sudalagunta, Silvia Regina Brandalise, Ana Carolina de Mattos Zeri, José Andrés Yunes

**Affiliations:** ^1^ Centro de Pesquisa Boldrini, Centro Infantil Boldrini, Campinas, SP, Brazil; ^2^ Department of Cancer Physiology, H. Lee Moffitt Cancer Center & Research Institute, Tampa, FL, United States; ^3^ Brazilian Biosciences National Laboratory (LNBio), Brazilian Center for Research in Energy and Materials (CNPEM), Campinas, SP, Brazil; ^4^ Medical Genetics Department, Faculty of Medical Sciences, State University of Campinas, Campinas, SP, Brazil

**Keywords:** acute lymphoblastic leukemia, methotrexate, glutathione, metabolomics, drug resistance, arsenic trioxide, thioredoxin reductase

## Abstract

**Introduction:**

Methotrexate (MTX), a folic acid antagonist and nucleotide synthesis inhibitor, is a cornerstone drug used against acute lymphoblastic leukemia (ALL), but its mechanism of action and resistance continues to be unraveled even after decades of clinical use.

**Methods:**

To better understand the mechanisms of this drug, we accessed the intracellular metabolic content of 13 ALL cell lines treated with MTX by 1H-NMR, and correlated metabolome data with cell proliferation and gene expression. Further, we validated these findings by inhibiting the cellular antioxidant system of the cells in vitro and in vivo in the presence of MTX.

**Results:**

MTX altered the concentration of 31 out of 70 metabolites analyzed, suggesting inhibition of the glycine cleavage system, the pentose phosphate pathway, purine and pyrimidine synthesis, phospholipid metabolism, and bile acid uptake. We found that glutathione (GSH) levels were associated with MTX resistance in both treated and untreated cells, suggesting a new constitutive metabolic-based mechanism of resistance to the drug. Gene expression analyses showed that eight genes involved in GSH metabolism were correlated to GSH concentrations, 2 of which (gamma-glutamyltransferase 1 [GGT1] and thioredoxin reductase 3 [TXNRD3]) were also correlated to MTX resistance. Gene set enrichment analysis (GSEA) confirmed the association between GSH metabolism and MTX resistance. Pharmacological inhibition or stimulation of the main antioxidant systems of the cell, GSH and thioredoxin, confirmed their importance in MTX resistance. Arsenic trioxide (ATO), a thioredoxin inhibitor used against acute promyelocytic leukemia, potentiated MTX cytotoxicity in vitro in some of the ALL cell lines tested. Likewise, the ATO+MTX combination decreased tumor burden and extended the survival of NOD scid gamma (NSG) mice transplanted with patient-derived ALL xenograft, but only in one of four ALLs tested.

**Conclusion:**

Altogether, our results show that the cellular antioxidant defense systems contribute to leukemia resistance to MTX, and targeting these pathways, especially the thioredoxin antioxidant system, may be a promising strategy for resensitizing ALL to MTX.

## Introduction

Acute lymphoblastic leukemia (ALL) is a subtype of leukemia that affects B- or T-cell precursor cells. It is the most common type of cancer in children, accounting for one quarter of all childhood cancer cases (1). Although the cure rates of childhood ALL have reached 85% in developed countries (2, 3), drug resistance is considered the major cause of treatment failure; as so, it is an issue of continuous and intense research (4–7).

Methotrexate (MTX), a folic acid competitor (antifolate) first synthesized in 1947, has been widely used as a chemotherapeutic agent and immune system suppressor. MTX is a cornerstone drug in ALL therapy. Once internalized by the cell through the folate receptor (RFC1), MTX undergoes sequential glutamylation by folyl-polyglutamate synthase (FPGS), thus becoming MTX polyglutamates (MTX-PG_2-6_). The molecule with a 2-6 glutamic residues tail is the active version of the drug; although its chemical activity is similar to the original drug, MTX-PG_2-6_ is preferentially kept in the intracellular environment [whereas non-glutamylated MTX is quickly exported by ABC transporters (8)], and cellular sensitivity to MTX is directly related to the intracellular pool of MTX-PGs (9). Recently, it was shown that ALL subtypes are associated with differential accumulation of MTX-PG levels: ALL harboring TCF3-PBX1 or ETV6-RUNX1 fusions presented lower MTX-PG levels, whereas hyperdiploid and BCR-ABL-like ALL had higher MTX-PG levels than other subtypes (10).

MTX primarily acts by inhibiting dihydrofolate reductase (DHFR), which catalyzes the sequential conversion of folic acid to dihydrofolate (DHF) and tetrahydrofolate (THF). Once folic acid is a precursor in *de novo* synthesis of thymidine, MTX imbalances nucleotide pools, ultimately leading to incorrect DNA synthesis and halting cell division. A high percentage of cells in S-phase is associated with a better clinical response to MTX (11) and is accompanied by increased expression of genes involved in nucleotide biosynthesis, such as *TYMS*, *CPTS*, and an MTX target enzyme, *DHFR (* 11). MTX also inhibits the bifunctional purine biosynthesis protein PURH (ATIC), blocking the conversion of the aminoimidazole carboxamide ribonucleotide (AICAR) into inosine monophosphate (IMP), thus inhibiting purine synthesis and the production of adenosine. AICAR accumulation leads to decreased one-carbon metabolism gene expression, cell proliferation, and increased global bioenergetic capacity *via* AMPK activation (12). Alternatively, MTX’s mechanism of resistance has been attributed to decreased MTX uptake due to deficiencies in membrane transport (13–15); increased expression of MTX efflux transporters (16–18); deficient polyglutamylation of MTX by FPGS (18–20); altered levels or structures of target enzymes (21, 22); and increased hydrolysis of MTXPGs by lysosomal gamma-glutamyl hydrolases (GGH) (23–25), although the influence of GGH on MTX efficacy remains controversial (26–28).

In parallel with MTX’s most well-known targets, several NAD+-dependent enzymes were inhibited by the antifolate *in vitro*: 2-oxoglutarate, isocitrate, malate, pyruvate, succinate, 6-phosphogluconate and glucose-6-phosphate dehydrogenases, glutamate-cysteine ligase, glutathione reductase, and glutathione peroxidase (29). MTX was shown to inhibit methionine S-adenosyltransferase (MAT) gene expression, protein levels amounts and activity *in vitro* and *in vivo*, resulting in decreased levels of S-adenosylmethionine (SAM) (30), the main methyl donor of metabolism.

Despite the number and importance of such enzymes to cellular metabolism, few studies have been conducted to better understand the effects of MTX on cellular metabolic homeostasis or find metabolic fragilities that could be exploited clinically. For instance, the depletion of multiple genes belonging to the histidine catabolism pathway increases resistance to MTX (31). This is caused by histidine catabolism draining the cellular pool of THF, which is already low in MTX-treated cells. The same study showed that *in vivo* dietary supplementation of histidine increased THF consumption by the histidine degradation pathway and enhanced the sensitivity of leukemia xenografts to MTX.

The antimetabolite nature of MTX and its inhibitory effect on many important metabolic enzymes prompted us to study the consequences of MTX treatment on cellular metabolism. We aimed to discover novel metabolic vulnerabilities that could be exploited therapeutically. Using a panel of 13 ALL cell lines (6 B-cell precursors and 7 T-cell ALL precursors) and untargeted hydrogen nuclear magnetic resonance (^1^H-NMR)-based metabolomics, we characterized MTX-triggered metabolic disturbance in detail. Though short term (48 hours) resistance to MTX seemed to be directly related to cellular proliferation, resistance in longer exposure times (96 hours) was associated with glutathione (GSH) levels. Gene expression analysis and pharmacological modulation confirmed the relevance of the cellular antioxidant system to MTX-mediated cell death susceptibility. Finally, we showed that pharmacological inhibition of the thioredoxin system by the chemotherapeutic drug arsenic trioxide (ATO) enhanced MTX cytotoxicity *in vivo* against one out of four primary human ALL cells, suggesting a novel therapeutic rationale to be further explored against ALL.

## Material and Methods

### Cell lines

Six B-cell precursor (Nalm6, Nalm16, Nalm30, REH, RS4;11, and 697) and 7 T-ALL (ALL-SIL, CCRF-CEM, HPB-ALL, Jurkat, Molt-4, P12-ICHIKAWA and TALL-1) cell lines were used in the study. The cell lines REH, 697, and RS4;11 were kindly provided by Dr. Sheila A. Shurtleff from St. Jude Children’s Research Hospital, Memphis, TN, USA; the cell lines Nalm16 and Nalm30 were provided by Dr. Akira Harashima from Hayashibara Biochemical Lab, Fujisaki, Japan; the cell lines ALL-SIL, HPB-ALL, P12-ICHIKAWA, and TALL-1 were kindly provided by Dr. João Barata from the Instituto de Medicina Molecular, Faculdade de Medicina da Universidade de Lisboa, Lisbon, Portugal; and the cell lines Jurkat, Nalm-6, CCRF-CEM (CEM) and Molt-4 were kindly provided by Dr. Alexandre E. Nowill from the State University of Campinas, Campinas, Brazil. Cell lines were cultured in RPMI-1640 culture medium (Cultilab, Campinas, Brazil); supplemented with 10% fetal bovine serum (Cultilab), 100 IU/ml of penicillin, and 100 μg/ml of streptomycin (Sigma-Aldrich, Saint Louis, MO, USA); and maintained at 37°C with a 5% CO_2_ atmosphere in all experiments.

### Reagents

All compounds were purchased from Sigma-Aldrich, resuspended according to the manufacturers’ instructions, and stored at –20°C. Subsequent dilutions used in the experiments were prepared at the moment of use.

### Cell viability assays

Eighty microliters of cell suspension (4 × 10^5^ cells/ml) were seeded in a 96-well cell culture plate, followed by the addition of 20 μl of drug solution in culture medium. Each dose was tested in triplicate. Negative controls received only vehicle, whereas positive (death) controls were added with 2 μl of an 18% paraformaldehyde solution immediately before viability determination. After the treatment period, the culture medium of each well was replaced by 0.2 ml of calcein AM solution (Sigma-Aldrich) at 2 μM in phosphate buffer [PBS 1X] and the culture plates were put into the incubator for 30 minutes before fluorescence reading using a Synergy H1 microplate reader (Biotek, Winooski, VT, USA; excitation/emission: 492/518 nm). To account for heterogeneous cell distribution within each well, fluorescence was measured in 25 points (a 5 × 5 matrix layout), with values then integrated (summed) at the end. Alternatively, the MTT (thiazolyl blue tetrazolium bromide; Sigma-Aldrich) reduction test was also used. In those cases, after 4 hours of incubation, 20 μl of MTT (5 mg/ml) were added to each well for cell metabolization. After overnight incubation, 0.1 ml of a dodecyl sulfate solution (10%) containing HCl (10 mM) was added to the wells. Absorbance was measured at 570 nm. Cell viability was expressed in relation to controls. Given that almost identical IC50 values for MTX were obtained by MTT and calcein AM at 48 and 96 hours, either one or the other method was used to access cell viability, depending on experimental and laboratory logistics.

### Determination of the doubling time

Two hundred microliters of a 2.5 × 10^5^ cell/ml cell suspension were seeded in a 96-well culture plate in triplicates. Fifteen microliters of the cell suspension were collected daily for cell count under the microscope (exclusion by trypan blue). The doubling time of each cell line was determined from the proliferation curves obtained.

### Cell treatment and metabolites extraction for ^1^H-NMR

Two hundred million cells in the exponential growth phase were suspended in culture medium (200 ml) and equally divided into 2 cell culture flasks. After 12 hours of acclimatization, either MTX (25 nM) or vehicle were added to the flasks. The doses were representative of the average MTX IC50 (96 hours) across all cell lines (18.4 nM). Following 24 hours of treatment, cells were washed with phosphate buffered saline (PBS 1X) and stored at –80°C. Both conditions (treatment and control) were analyzed in 3 biological triplicates for each cell line. The intracellular polar metabolic content was obtained following the methanol/chloroform extraction protocol, adapted from La Belle (32). Briefly, cells were disrupted (Sonics Vibra Cell, Sonics & Materials Inc., Newtown, CT, USA) in an ice-cold mixture of methanol (1.66 ml) and chloroform (0.83 ml) for 3 minutes. Next, ice-cold Milli Q water (1.25 ml) and chloroform (1.25 ml) were added to the sonicated mixture, followed by rapid vortexing. After centrifugation (3.4 × 10^3^ g for 20 minutes at 4°C), 2.6 ml of the supernatant phase (containing water, methanol, and polar metabolites) were collected and freeze-dried.

### Acquisition of ^1^H-NMR spectra and metabolic profiling

The lyophilized metabolic extracts were suspended in 0.6 ml of deuterated water (Cambridge Isotope Laboratories Inc., Tewksbury, MA, USA) containing phosphate buffer (100 mM) and TSP (3-3-(trimethylsilyl)-2,2,3,3-tetradeuteropropionic acid or TMSP-*d*_4_, Cambridge Isotope Laboratories) (0.5 mM) and inserted into a 5 mm NMR tube for immediate acquisition. Spectra were acquired on a Varian Inova NMR spectrometer (Agilent Technologies Inc., Santa Clara, CA, USA), equipped with a triple resonance probe and operating at a resonant frequency of ^1^H at 500 MHz and a constant temperature of 25°C. Two hundred and fifty-six scans were performed with delays of 1.5 seconds, a reading window of 16 ppm, and an acquisition time of 4 seconds. Phase and baseline corrections, as well as metabolic identification and quantification, were performed in Chenomx NMR Suite 7.1 (Chenomx Inc., Edmonton, Canada). In total, 78 ^1^H-NMR spectra were analyzed. Metabolite set enrichment analyses were performed with the platform MetaboAnalyst (33) (http://www.metaboanalyst.ca).

### Gene expression analysis

Cell lines (5 to 10 × 10^6^ cells) in the exponential growth phase were maintained overnight in fresh culture medium and then had their RNA extracted using the Illustra RNAspin Mini Kit (GE Healthcare Life Sciences, Pittsburgh, PA, USA). Samples were processed with the One-Cycle Target Labeling and Control Reagents Kit (Affymetrix, Santa Clara, CA, USA) and hybridized on HG-U133 Plus 2.0 Arrays (Affymetrix). Expression values were obtained with the iterPLIER+16 algorithm and expressed in a log2 scale. For additional details, please see Silveira et al. (34). Gene set enrichment analyses (GSEA) (http://www.broadinstitute.org/gsea/) were performed with 1,000 gene set permutation. Phenotypic labels (eg, GSH levels or MTX IC50) were used as continuous labels. The GSEA platform used Pearson’s correlation to determine the degree of linear relationship between gene sets and phenotypes. Only probe sets/transcript clusters annotated with a Gene Symbol were used in the analyses. Gene sets were considered significantly enriched when *P* ≤ 0.05 and FDR ≤ 0.05.

### High-throughput *in vitro* drug and combination screening assay

A high-throughput *in vitro* drug screening assay was used to quantify the chemosensitivity of leukemia cell lines as described previously (35, 36). Briefly, leukemia cells were seeded in 384-well, black, optically clear flat-bottom PhenoPlate (PerkinElmer, Waltham, MA) with collagen I (PureCol, Advanced Biomatrix, Carlsbad, CA) to a total volume of 9 μl, containing approximately 3,000 leukemia cells in each well. After collagen polymerization (1 hour at 5% CO_2_ and 37°C), each well was filled with 73 μl of culture media and left overnight in the incubator for cell acclimatization. In the next day, 9 μl of the drug dilutions (BSO, Auranofin, BSO+Auranofin) were added to the wells so that every drug/combination was tested at 5 (fixed concentration ratio, for combinations) concentrations (1:3 serial dilution) in 2 replicates. Negative controls containing vehicle (DMSO, maximum concentration of 0.5%) were included. Plates were placed in a motorized stage microscope (EVOS Auto FL 2, Thermo Fisher Scientific, Waltham, MA) equipped with an on-stage incubator and maintained at 5% CO_2_ and 37°C. Each well was imaged every 30 minutes for a total duration of 96 hours. A digital image analysis algorithm (37) was implemented to determine changes in viability of each well longitudinally across the 96-hour interval. This algorithm computes differences in sequential brightfield images and identifies live cells with continuous membrane deformations resulting from their interaction with the surrounding extracellular matrix. These interactions cease upon cell death. By applying this operation to all images acquired for each well, we were able to quantify nondestructively the effect of the drugs and their combinations as a function of concentration and exposure time – ie, by area under the curve (AUC) and lethal dose for median effect (LD50). Cell line-specific doses were chosen based on the MTT reduction assay and on previous standardization experiments. Synergy was determined by using the method described by Sudalagunta et al (38), in which percent live cells across time and the serially diluted drug(s) concentrations are used to compute additive response using the Bliss Independence Model. The additive response serves as a reference to determine the extent of synergy observed in each cell line by comparing it with the percent live cells measured when treated with the combination (at a fixed ratio of the 2 constituent single agents). The metric used to quantify *in vitro* drug synergy is given by the log_2_ ratio of the additive LD50 (computed from the two single agent responses as described above) and the combination LD50 for each cell line. This *in vitro* drug sensitivity characterization and combination effect framework was developed and pioneered by Dr Ariosto S. Silva’s group (Moffitt Cancer Center), which resulted in several significant contributions in the field of hematological malignancies over the past five years (39–42).

### Calcein AM for cell viability analysis by flow cytometry

Cells were seeded in 96 well plates at 3.0e5 cells/well in 100 µl of RPMI-1640 with 10% FBS. Methotrexate was added at different concentrations in triplicates. After 48 hours of incubation, a fixed dose of Arsenic Trioxide was added and 48 hours later cell viability was measured by the MTT method. To confirm the MTT results, a replicate plate run in parallel was developed by adding 5 µl of 2.5 µM Calcein-AM (ThermoFisher Scientific) to selected wells and incubated 15 min at room temperature. The number of viable cells was measured by flow cytometry after addition of 5 µl spike in fluorescently labeled bead (CountBright™ Plus Absolute Counting Beads, ThermoFisher Scientific) for normalization. The number of Calcein-positive cells per 1.0e3 beads was computed. Some of the cell lines that were more sensitive to the drug combination were assayed in 48-hour experiments instead of 96 hours. In this case, ATO was added after the first 24 hours of treatment with MTX and the number of viable cells was measured 24 hour later. In this case, cells were cultured in 24-well plates at 1.2e5 cells/well in 500 µl of RPMI-1640 with 10% FBS. At the end of the experiment (48 hours), 100 µl were transferred to a cytometer tube, 0.5 µl of 25 µM Calcein-AM were added and, after 15 min of incubation at room temperature, 5 µl of fluorescently labeled beads plus 195 µl of PBS 1X were added and cells were analyzed by flow cytometry. Flow cytometry was performed on a LSRFortessa X-20 (Becton Dickinson, Franklin Lakes, NJ) and data were analyzed using FlowJo (v7.1.6, TreeStar).

### *In vivo* efficacy of ATO+MTX against primary ALL

Cryopreserved primary ALL cells obtained from a mouse of the IL7R^<CPT>^ knock-in model (43) or from 4 pediatric ALL patients (2 BCP- and 2 T-ALL) were used in this study. Cells were thawed, washed in PBS 1X buffer, and injected into nonirradiated mice *via* the tail vein. Sick animals were killed and had their spleen macerated, and ALL cells were obtained by Ficoll gradient centrifugation. Fresh ALL cells were then transplanted into more mice for the experiments. The murine ALL was transplanted into C57BL/6 mice. Treatment started 2 days after transplantation. Mice (n = 12) were randomly distributed into 4 treatment groups: vehicle (PBS 1X with 1% NaOH), arsenic trioxide (ATO; 2.5 mg/kg), methotrexate (MTX; 1, 5, or 10 mg/kg), and ATO+MTX. Drugs were intraperitoneally administered once a day, 5× per week. When in combination, MTX was given 6 hours before ATO (based on Szymanska et al (44), who used this interval for other drug combinations). Overall survival was measured from after 2 days the date of leukemia injection to the date of animal death or when humane endpoint criteria were reached. Differences in survival were determined by Log-rank (Mantel-Cox) test using the GraphPad Prism v7.0 (GraphPad, La Jolla, CA) software. The study was approved by the Animal Experimentation Ethics Committees of University of Campinas (CEUA/UNIMCAP, protocol 4557-1(A)/2018). Experiments with the human ALLs were carried out in nonirradiated NSG (NOD.Cg-PrkdcscidIl2rgtm1Wjl/SzJ) mice (Jackson Laboratory). Leukemia progression was monitored weekly in peripheral blood post-Ficoll mononuclear cells by flow cytometry using anti-human CD45-APC (clone HI30, ImmunoTools) and anti-mouse CD45-BV605 (clone 30-F11, BD Horizon). Flow cytometry was performed on a LSRFortessa X-20 and data were analyzed using FlowJo. When hCD45^+^ cells reached 0.1% of total CD45^+^ peripheral blood cells in half of the mice (n = 24), animals were randomly distributed among the 4 treatment groups. T-ALL PDXs were treated with vehicle, ATO (2.5 mg/kg), MTX (5 mg/kg for BCP-ALL; 10 mg/kg for T-ALL) or ATO+MTX, as described above. Treatment lasted for 4 weeks and was administered in consecutive weeks for T-ALL xenografts or every other week for BCP-ALL (the BCP-ALL cells used were more sensitive to MTX than T-ALL). Death by acute toxicity was censored. Overall survival was measured from the date of leukemia engraftment to either the animal death, when the proportion of hCD45^+^ cells in the peripheral blood > 25% (45), or when humane endpoint criteria were reached. The study was approved by the Animal Experimentation Ethics Committees of Centro Infantil Boldrini (CEUA/BOLDRINI, protocol 0016-2020) and Human Ethics Committees of Centro Infantil Boldrini (CAAE 34601120.7.0000.5376).

### Statistical analyses

Paired nonparametric Wilcoxon signed rank tests and other analyses and graphs (dose-response curves, estimates of IC50 values, correlation plots, Kaplan-Meier curves, and log-rank tests for survival analyses) were made in Prism v7.0 (GraphPad, La Jolla, CA, USA). *P* ≤ 0.05 was considered significant.

## Results

### MTX impact on cellular metabolism

Initially, cells were treated with increasing doses of MTX for 48 or 96 hours to determine their IC50. IC50 at 48 hours ranged from 10.8 to 85.5 nM in T-ALL and from 14.7 to 101.1 nM in BCP-ALL. IC50 at 96 hours ranged from 5.5 to 38.4 nM in T-ALL and from 9.1 to 31.7 n in BCP-ALL (**Supplementary Figure 1**).

To determine the impact of MTX on cell metabolism, cell lines underwent unsupervised global metabolic profiling by ^1^H-NMR in either the presence or in the absence of the drug (a representative ^1^H-NMR spectrum is shown in **Supplementary Figure 2**). All cell lines were treated with a fixed dose of MTX (25 nM) for 24 hours, as this dose was representative of MTX’s average IC50 at 96 hours across all cell lines (ie, 18.4 nM). By treating the cells with the same dose of MTX, we intended to find cell line-specific metabolic differences under the same drug challenge. We reasoned that using cell-specific IC50 doses of MTX would have overthrown any metabolic differences among cell lines. This approach is in line with previous metabolomic studies that have adopted a similar rationale (46, 47). Additionally, this condition elicited the greatest metabolic variance, compared to high-dose MTX and shorter treatment time, in a pilot study on a MTX-resistant *vs*. a MTX-sensitive cell line (**Supplementary Figure 3**).

**Figure 1:**
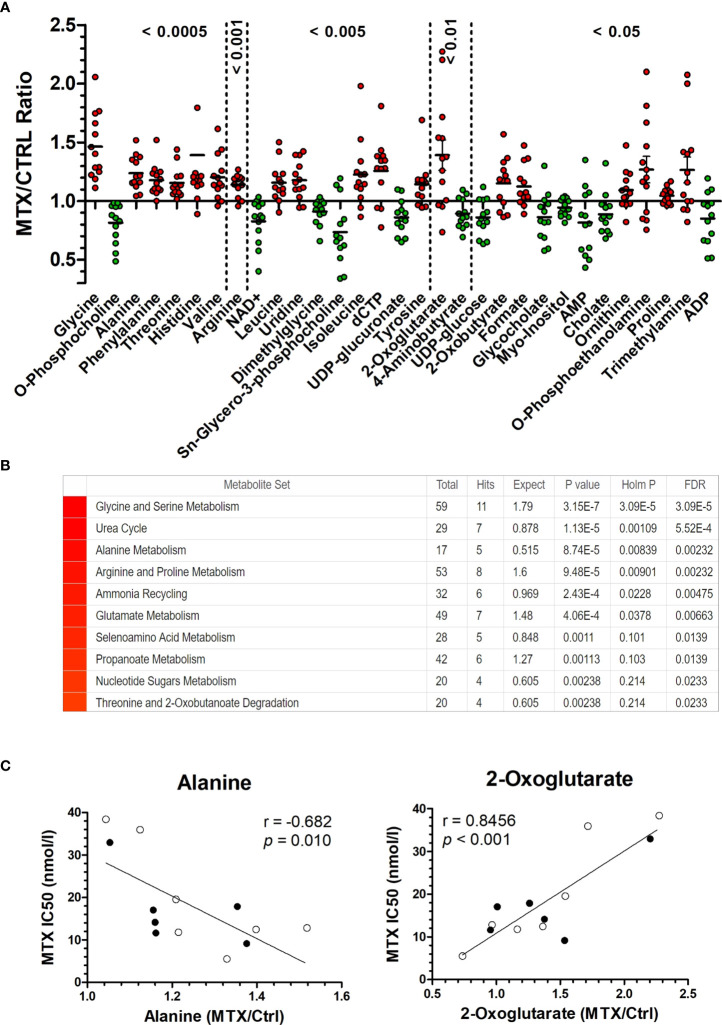
MTX-driven metabolic perturbations in ALL. **(A)** Metabolic disturbance caused by MTX treatment. Each dot is the mean of three biological replicates for each cell line. Metabolites’ concentrations fold change (in MTX-treated/untreated cells) above 1 indicate increase caused by MTX, whereas values below 1 indicate antifolate-induced decrease (*P*-values, Wilcoxon signed ranked test, after comparison of the distributions with the hypothetical mean value of 1). **(B)** Enrichment score for metabolites in **(A)** indicate the metabolic pathways and processes most impacted by MTX. **(C)** Correlation between metabolite concentration in untreated cells and MTX resistance (IC50 at 96 hours). Black and white beads represent BCP-ALL and T-ALL cell lines, respectively. *P*-values and *r* are from Pearson correlation.

In total, 70 metabolites were identified and quantified. Metabolites’ identifiers, concentrations, and fold changes across all samples can be found in **Supplementary Tables 1–3**, respectively. We found that 19 metabolites were significantly increased by MTX, whereas 12 were decreased in response to the drug treatment (**Figure 1A** and **Supplementary Table 4**). Several amino acids had their concentrations increased following MTX treatment: glycine, alanine, phenylalanine, threonine, histidine, valine, arginine, leucine, isoleucine, tyrosine, and proline. 2-oxobutyrate, a byproduct of the conversion of cystathionine into cysteine and first intermediate in threonine degradation, was also increased with drug treatment. MTX treatment produced effects consistent with alterations in the folate cycle (formate); pyrimidine (uridine and dCTP) and purine (AMP, ADP, and NAD+) nucleotide synthesis; the pentose phosphate pathway (UDP-glucuronate, UDP-glucose, and myo-inositol); phospholipid synthesis (phosphocholine, sn-glycero-3-phosphocholine, phosphoethanolamine, and trimethylamine); the citric acid cycle (2-oxoglutarate); and the uptake of bile acids (cholate and glycocholate).

A metabolite set enrichment analysis made from the 31 metabolites significantly modulated by MTX confirmed the major impact of the drug on glycine and serine metabolism, the urea cycle, alanine metabolism, and arginine and proline metabolism (**Figure 1B**). A scheme integrating all MTX-modulated metabolites into a single metabolic panel can be found in **Supplementary Figure 4**. Interestingly, a few cases of differential metabolic changes (ie, fold change of MTX treatment over basal levels) were associated with MTX resistance (at 96 hours); for example, the greatest fold changes for alanine were observed among the most sensitive cells to MTX, whereas 2-oxoglutarate (α-ketoglutarate) greatest fold changes were found among the most resistant cells to the antifolate (**Figure 1C**). Although a causal relationship cannot be established, this finding indicates that MTX resistance *in vitro* is associated with specific metabolic responses by the cells under drug action.

### Metabolic levels associated with MTX resistance

**Figure 2:**
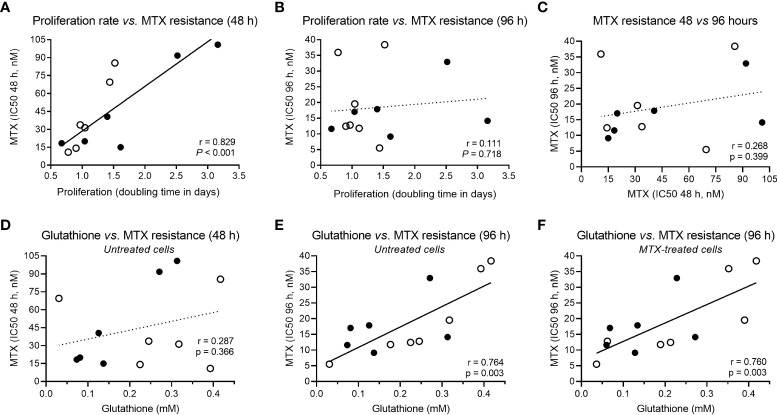
Correlation between cellular proliferation, MTX resistance and GSH levels. **(A)** Correlation between proliferation rate (cell doubling time) and MTX resistance (IC50) at 48 hours or **(B)** 96 hours, and **(C)** across MTX resistance over time (48 × 96 hours). **(D)** Correlation between constitutive GSH and MTX resistance at 48 hours or **(E)** 96 hours, and **(F)** between GSH levels in MTX-treated cells and MTX IC50 at 96 hours. Black and white beads represent BCP-ALL and T-ALL cell lines, respectively. *P*-values and *r* are from Pearson correlation.

In addition to the association between metabolic fold change and MTX resistance, we searched for correlations between metabolic concentration and resistance to MTX (expressed as IC50) at 48 and 96 hours in either the presence or absence of the drug (**Table 1**). Given that MTX is more effective against dividing cells because of its effect on the nucleotide synthesis, we also examined correlations between metabolites concentrations and cell proliferation rate, as this could be a confounding factor (proliferation curves for doubling time determination are in **Supplemental Figure 5**). In fact, MTX resistance at 48 hours and proliferation rate were tightly correlated (Pearson r = 0.829, *P* < 0.001) (**Figure 2A**); however, the association was lost for drug resistance at 96 hours (Pearson r = 0.111, *P* = 0.718) (**Figure 2B**), and MTX resistance at 48 hours was not associated with resistance at 96 hours (Pearson r = 0.268, *P* = 0.399) (**Figure 2C**), indicating that factors unrelated to cell proliferation were contributing to cellular resistance to MTX during longer exposure times.

**Table 1:** Correlation between the concentration of selected metabolites and cell proliferation (expressed in terms of doubling time, in days) or MTX resistance (at 48 or 96 hours) in cells either treated or not with MTX.

Metabolite	Proliferation rate (doubling time)				MTX IC50 (48 hours)				MTX IC50 (96 hours)			
	Untreated cells		MTX-treated cells		Untreated cells		MTX-treated cells		Untreated cells		MTX-treated cells	
	Pearson r	P	Pearson r	P	Pearson r	P	Pearson r	P	Pearson r	P	Pearson r	P
2-Methylglutarate	-0.521	0.068	-0.339	0.257	-0.541	0.056	-0.512	0.074	0.248	0.415	0.064	0.836
Acetate	-0.506	0.078	-0.354	0.236	**-0.637**	**0.019**	**-0.608**	**0.028**	-0.120	0.697	-0.320	0.286
AMP	-0.228	0.454	-0.194	0.526	-0.479	0.098	-0.336	0.262	-0.436	0.136	-0.265	0.381
Asparagine	-0.141	0.647	-0.063	0.838	-0.285	0.345	-0.242	0.425	**0.602**	**0.030**	0.517	0.070
Carnitine	-0.455	0.118	-0.196	0.522	**-0.754**	**0.003**	**-0.569**	**0.043**	-0.060	0.846	0.081	0.793
Cholate	-0.548	0.053	-0.373	0.209	**-0.614**	**0.026**	-0.402	0.174	0.198	0.516	0.337	0.261
Creatine	**0.619**	**0.024**	**0.620**	**0.024**	0.325	0.279	0.356	0.233	0.291	0.335	0.290	0.337
Cytidine	**-0.609**	**0.027**	-0.434	0.139	-0.536	0.059	-0.420	0.153	-0.191	0.531	-0.212	0.487
Formate	**-0.821**	**0.001**	**-0.634**	**0.020**	**-0.814**	**0.001**	**-0.836**	**0.000**	-0.267	0.378	-0.540	0.057
Glutathione	0.217	0.477	0.258	0.395	0.161	0.600	0.186	0.543	**0.663**	**0.014**	**0.672**	**0.012**
GTP	-0.357	0.231	-0.197	0.518	-0.501	0.081	-0.253	0.404	-0.253	0.405	-0.054	0.860
Guanosine	0.436	0.136	0.488	0.090	0.368	0.216	0.527	0.064	**0.554**	**0.049**	0.449	0.123
Hypoxanthine	-0.427	0.145	-0.303	0.314	**-0.562**	**0.046**	-0.383	0.196	-0.501	0.081	-0.341	0.254
Lactate	-0.343	0.252	-0.142	0.644	-0.510	0.075	-0.304	0.312	-0.137	0.655	-0.055	0.859
Malate	-0.483	0.095	-0.264	0.384	**-0.671**	**0.012**	-0.494	0.086	-0.112	0.716	-0.078	0.801
myo-Inositol	**0.647**	**0.017**	**0.633**	**0.020**	0.357	0.231	0.332	0.268	0.169	0.581	0.188	0.539
NAD+	0.469	0.106	0.511	0.075	0.524	0.066	0.535	0.060	0.122	0.691	0.137	0.657
NADP+	0.321	0.285	0.447	0.126	0.468	0.107	0.521	0.068	0.009	0.977	0.250	0.410
Pyroglutamate	-0.316	0.293	0.015	0.961	-0.520	0.069	-0.205	0.501	-0.062	0.841	-0.036	0.908
S-Adenosylhomocysteine	-0.419	0.155	**-0.554**	**0.049**	-0.487	0.091	**-0.669**	**0.012**	-0.072	0.814	-0.227	0.457
Succinate	-0.392	0.186	-0.421	0.152	**-0.668**	**0.013**	**-0.615**	**0.025**	-0.428	0.145	-0.330	0.270
**Other parameters**	**Pearson r**		**P-value**		**Pearson r**		**P-value**		**Pearson r**		**P-value**	
Proliferation rate	1.000		0.000		**0.827**		**0.001**		0.113		0.714	
MTX IC50 (48 h)	**0.827**		**0.001**		1.000		0.000		0.303		0.315	
MTX IC50 (96 h)	0.113		0.714		0.303		0.315		1.000		0.000	

Dark gray and bold, significant association (P < 0.05); light gray, 0.05 < P < 0.10.

Several metabolites were negatively associated with MTX resistance at 48 hours: acetate, carnitine, cholate, formate, hypoxanthine, malate, and succinate (**Table 1**). Acetate is the product of glycolysis, malate and succinate are citric acid cycle elements, carnitine is important for lipid beta-oxidation, and hypoxanthine is a purine derivative, suggesting that, at shorter exposure times (48 hours), MTX sensitivity is associated with increased cellular metabolic activity. Alternatively, MTX resistance at 96 hours was positively associated with asparagine, guanosine, and glutathione (GSH) levels (**Table 1**).

GSH is the main antioxidant of the cell and is important in preventing damage caused by reactive oxygen species (ROS). Interestingly, although not associated with MTX resistance at 48 hours (Pearson r = 0.287, *P* = 0.366) (**Figure 2D**), GSH was the only metabolite associated with MTX resistance in both untreated (Pearson r = 0.764, *P* = 0.003) (**Figure 2E**) and treated cells (Pearson r = 0.780, *P* = 0.003) (**Figure 2F**) at 96 hours, indicating a constitutive metabolic trait predictive of cellular resistance to the MTX insult. Its association with MTX resistance is in line with the fact that MTX is a ROS-generating drug (48, 49). Correlation coefficients between all metabolites concentrations, cell proliferation rate, and MTX resistance (at 48 and 96 hours) in treated and untreated samples are in **Supplementary Table 5**.

### GSH levels associated with the expression of GSH metabolism genes

**Figure 3:**
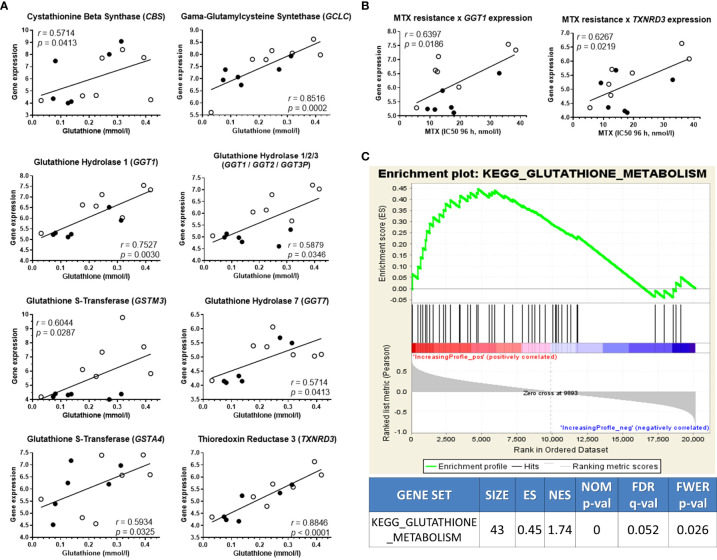
GSH-related transcriptome associates with metabolic levels and MTX resistance. **(A)** Association between glutathione (GSH) metabolism genes’ expression and GSH concentration; **(B)** Gene set enrichment analysis score plot showing KEGG’s Glutathione Metabolism gene set enrichment in high-GSH ALL cell lines. **(C)** Expression of two GSH-related genes also correlated to MTX resistance. Black and white beads represent BCP-ALL and T-ALL cell lines, respectively. *P*-values and *r* are from Pearson correlation.

We then searched for genetic traits that could explain the metabolite variability among the cell lines. For this purpose, the concentration of asparagine, guanosine, and GSH was correlated to the expression of genes belonging to their respective metabolic pathways. Though no associations for asparagine or guanosine-related genes were found, 8 genes that code for enzymes that participate in the GSH metabolism were positively correlated to GSH levels: *CBS*, *GCLC*, *GSTM3*, *GSTA4*, *GGT1*, *GGT1*/*GGT2*/*GGT3P*, *GGT7*, and *TXNRD3* (**Figure 3A**). The expression of all genes involved in asparagine, guanosine, and GSH metabolisms that did not correlate to their respective metabolite’s levels are in **Supplementary Figure 6**. Interestingly, 2 of the GSH metabolism genes that correlated with GSH levels (*GGT1* and *TXNRD3*) were also associated with MTX resistance (**Figure 3B**). Moreover, a Gene Set Enrichment Analysis (GSEA) confirmed that the expression of KEGG’s “Glutathione Metabolism” gene set (**Supplementary Table 6**) was enriched in MTX-resistant samples (**Figure 3C**). These findings offer a genetic basis for the differential concentration of GSH across the samples with distinct MTX sensitivity.

### Promoting GSH metabolism induces proliferation and MTX resistance

**Figure 4:**
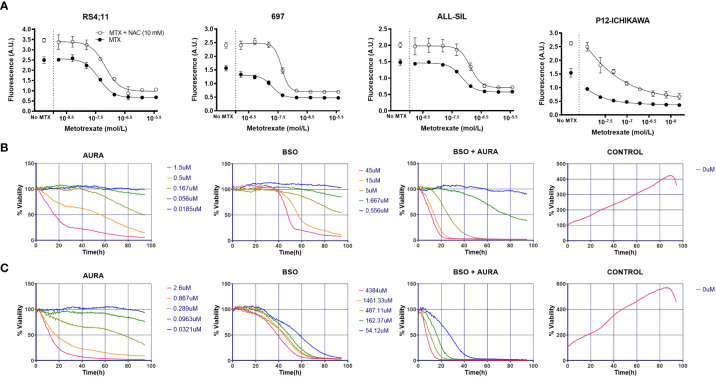
*In vitro* combination of GSH pathway modulators with MTX or a thioredoxin reductase inhibitor. **(A)** Survival curves to increasing doses of MTX in either the presence or absence of NAC in four representative cell lines, accessed by the calcein AM method after 96 hours of treatment. A.U. = arbitrary units. **(B)** BCP-ALL RS4;11 survival in response to auranofin (AURA), buthionine sulphoximine (BSO), BSO + AURA and vehicle (CONTROL) accessed by bright field imaging (high-throughput drug combination screening assay) over 96 hours. **(C)** Idem **(B)**, for TALL-1 cell line. In the BSO + AURA plots, curve colors indicate the corresponding doses of the single agents that were combined.

Given that both metabolic and gene expression data pointed to GSH metabolism as an important factor contributing to MTX resistance in ALL cells, we evaluated the effect of either enhancing or inhibiting GSH metabolism on cellular resistance to MTX. Initially, we treated the cell lines with increasing doses of MTX in the presence of N-acetylcysteine (NAC), a precursor of the *de novo* synthesis of GSH that is converted into cysteine by glutamate-cysteine ligase (GCL). We observed that NAC not only protected cells from MTX cytotoxicity but also promoted cell proliferation, including in RS4;11, 697, ALL-SIL and P12-ICHIKAWA (**Figure 4A**) — 4 cell lines with relatively low constitutive GSH levels — whereas cell lines with relatively high GSH (eg, HPB-ALL and Jurkat) tended to present less pronounced effects. The graphs for all cell lines are in **Supplementary Figure 7**.

### Inhibiting GSH metabolism does not sensitize cells to MTX

We also treated leukemia cells with increasing doses of 4 interferents of GSH metabolism: phenethylisothiocyanate (PEITC) and piperlongumine (PL), which deplete GSH and increase ROS (50–52); hydrogen peroxide (H_2_O_2_), a ROS itself; and buthionine sulphoximine (BSO), a GCL inhibitor and, thus, a pharmacological inhibitor of GSH synthesis. The IC50 obtained for all these compounds across the cell lines are listed in **Supplementary Table 7**. Surprisingly, these ROS-promoting compounds did not enhance MTX cytotoxicity significantly (**Supplementary Figure 8**), even though we found a correlation between PL or H_2_O_2_ resistance and MTX resistance (**Supplementary Figure 9**). In sum, these results show that, while promoting GSH metabolism consistently increases leukemia resistance to MTX — sometimes even accompanied by simultaneous increase in proliferation — its inhibition does result in sensitization to the antifolate.

### Inhibiting the thioredoxin system potentiates MTX cytotoxicity *in vitro*

The intriguing asymmetrical contribution of GSH metabolism on MTX resistance suggested that a compensatory mechanism could be counterbalancing GSH metabolism inhibition and, thus, protecting cells from MTX insult.

As showed previously, thioredoxin reductases 3 (*TXNRD3*) expression was correlated to both intracellular GSH levels and MTX resistance (**Figures 3A, B**). TXNRD3 is a member of the cellular antioxidant arsenal whose main role is to chemically reduce thioredoxins, antioxidant proteins that facilitate the reduction of oxidized client proteins by cysteine thiol-disulfide exchange. Oxidized thioredoxin reductases, in turn, are reduced back to their functional state by NADPH. GSH and thioredoxin, therefore, are the 2 known antioxidant systems whose co-occurrence is crucial for the maintenance of the cellular redox homeostasis. We used a high-throughput single agent and drug combination screening assay based on bright-field images to determine cell survival under treatment with auranofin (Aura), a clinically approved thioredoxin reductase inhibitor for rheumatoid arthritis, and the GSH synthesis blocker BSO (**Figures 4B, C** and **Supplementary Figure 10**). A synergistic interaction between the drugs was observed in 5 out 6 cell lines tested using the method described by Sudalagunta et al (38), in which percent live cells across time and the serially diluted drug(s) concentrations are used to compute additive response using the Bliss Independence model (**Supplementary Table 8**). We confirmed the co-dependence of ALL cells on these antioxidant systems in 3 cell lines by the MTT viability assay (**Supplementary Figure 11**).

**Figure 5:**
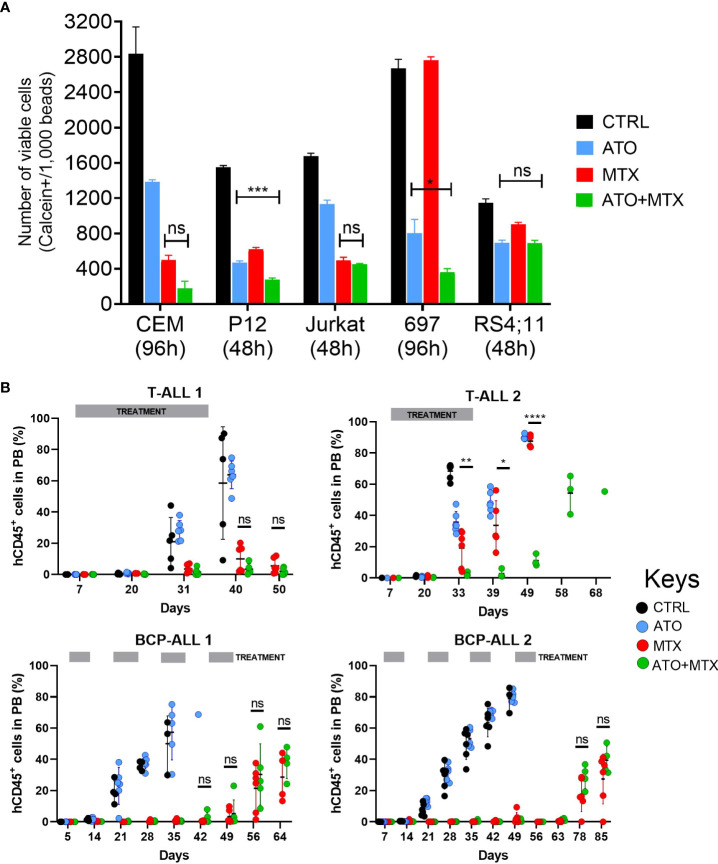
Combination of ATO and MTX *in vitro* and in PDX models of ALL. **(A)** Cell survival accessed by flow cytometry analysis of calcein-positive cells. The number of viable calcein+ cells per 1,000 fluorescent beads (spike in) was determined after 48 or 96 hours, as indicated. MTX was added at time zero while ATO was added at half-time. Results are the mean ± standard error (SE) of triplicate samples. *P*-values for Tukey’s Test following ANOVA. *P<0.05; ***P<0.001. Doses used: CCRF-CEM: ATO = 1.5 µM, MTX = 12.5 nM; P12-ICHIKAWA: ATO = 3 µM, MTX = 13 nM; Jurkat: ATO = 1.5 µM, MTX = 36 nM; RS4;11: ATO = 1.4 µM, MTX = 18 nM; 697: ATO = 0.75 µM, MTX = 6 nM. **(B)** Human CD45-positive (hCD45+) leukemia cells in peripheral blood of PDX-transplanted NSG mice, accessed by flow cytometry. Treatment started one week after engraftment when hCD45+ cells > 0.5% in half of the mice (Day 7) and was administered for 4 consecutive weeks against T-ALL or every other week against BCP-ALL. Doses used: MTX = 10 mg/kg daily (T-ALL) or 5 mg/kg daily (BCP-ALL); ATO = 2.5 mg/kg daily; ATO was administered 6 hours after MTX in the co-treatment cohort. L.E. = leukemia engraftment period. *P*-value for Log-rank test. *P*-value for unpaired t test between MTX and ATO+MTX. *P≤0.05; **P≤0.01; ****P≤0.0001; ns, not significant.

Next, we investigated the role of the antioxidant thioredoxin system on MTX sensitivity *in vitro*. For this purpose, we tested the effect of MTX in combination with 2 thioredoxin reductase inhibitors, arsenic trioxide (53) (ATO), and auranofin. We observed a mild potentiation of the MTX effect by ATO (**Figure 5A**) and Auranofin (**Supplementary Figure 12**) but only for few of the cell lines tested.

Altogether, our results indicate that the thioredoxin system exerts a compensatory effect over the GSH pathway that is not reciprocal and suggest that the thioredoxin system might be a potential target for MTX re-sensitization in leukemia cells.

### *In vivo* antileukemic effects of ATO and MTX in murine and patient-derived leukemia

Although mild, the antileukemic effects of the combinations between thioredoxin reductase inhibitors and MTX *in vitro* motivated us to test them *in vivo*. Here we chose the ATO+MTX combination given ATO’s current use in the clinic as a chemotherapeutic agent against acute promyelocytic leukemia. Our first investigation was using an immunocompetent mouse model transplanted with a primary murine B-ALL obtained from our *Il7r* knock-in mouse model (43). We tested different doses of MTX (1, 5, and 10 mg/kg) and found a dose-response pattern, with 10 mg/kg showing best results. ATO+MTX significantly increased overall survival compared to vehicle or single agents, though the difference was modest (**Supplementary Figure 13**).

Next, we tested the efficacy of the drug combination in 4 patient-derived xenografts (PDXs; 2 BCP-ALL and 2 T-ALL) *in vivo*. The PDXs represented a diverse genetic background: T-ALL 1 carries an activating *IL7R* mutation that confers a particular aggressive phenotype (54), and BCP-ALL 1 expresses the TCF3-PBX1 fusion protein as a result of the t(1;19) chromosomal translocation. Mice transplanted with T-ALL PDXs were treated with the same dose (10 mg/kg) and schedule as mice transplanted with murine ALL cells, whereas BCP-ALL PDX-transplanted mice received MTX at a lower dose (5 mg/kg) and were treated every other week to minimize the contribution of MTX to disease control and thus maximize ATO’s effect. However, this approach resulted in longer treatment periods and increased gastrointestinal toxicity with some fatalities that were censored in the analysis (**Supplementary Table 9**). Of the 4 PDXs tested, only one T-ALL was resistant to MTX (remission lasted for 20 days compared to ≥ 50 days in the other 3 PDXs). In this case, the ATO+MTX combination significantly decreased tumor burden in the peripheral blood (**Figure 5B**) and extended overall survival (**Supplementary Figure 14**) compared to vehicle alone or either drug used as a monotherapy. Altogether, these results suggest that MTX-resistant ALL patients could benefit from therapies that include thioredoxin reductase inhibitors in combination with MTX.

## Discussion

Seventy years after MTX was first synthesized, our comprehension of its mechanism of action and impact on cellular metabolism continues to expand. In this study, we explored the metabolic-wide changes caused by MTX in a panel of T- and B-cell precursors ALL cell lines, aiming to characterize the perturbations caused by the drug on the intracellular metabolome and provide new insights about metabolic vulnerabilities for future therapeutic exploitation.

We were able to identify and quantify 70 metabolites from diverse metabolic pathways; 19 were increased with drug treatment and 12 were decreased. Enrichment analyses showed that the metabolism of several amino acids was particularly impacted, highlighting the glycine and serine metabolism as the topmost altered pathway. Tedeschi et al (55) reported a 2-fold increase in glycine concentration in breast cancer cell lines treated with MTX, which is similar to what we observed in our study. We propose that glycine accumulation is the consequence of an MTX-induced halt in the glycine cleavage system (GCS). The GCS is main mechanism for glycine degradation and requires THF and NAD+ for glycine decarboxylation. MTX leads to THF shortage by inhibiting DHFR, and NAD+ was also decreased following MTX treatment (as seen in **Figure 1A**). Glycine can be derived from threonine, whose concentration was also increased in MTX-treated cells.

The pentose phosphate pathway (PPP) is an anabolic pathway diverted from glycolysis through glucose-6-phosphate dehydrogenase (G6PD), producing NADPH as well as ribose-5-phosphate, a precursor of purines biosynthesis. MTX was shown to inhibit G6PD *in vitro* (29). In our study, UDP-glucose, UDP-glucuronate, and myo-inositol, all intermediates of the PPP, were decreased after drug treatment, suggesting that G6PD inhibition in situ is also occurring and is an important feature of MTX action. Additionally, decreased levels of adenine, hypoxanthine, NAD+, AMP, ADP, and ATP also point towards ATIC inhibition — MTX’s main mechanism of action as an anti-inflammatory drug (56). Decreases in NAD+, AMP, ATP, and hypoxanthine levels were also observed in the erythroblastic cell line K562 following MTX treatment (57). Pyrimidine biosynthesis appears to be impaired by the antifolate as well. Thymidylate synthase (TS) was shown to be inhibited *in vitro* by MTX (58, 59); with TS inhibition, its substrate dUMP would be diverged from dTMP to dCMP synthesis, a precursor of uridine and dCTP — both of which were increased in our data set.

Decreases of several metabolites related to phospholipid metabolism (o-phosphocholine, sn-glycero-3-phosphocholine, phosphoethanolamine, and trimethylamine) indicated that MTX interferes with membrane synthesis. Lipid and membrane synthesis impairment has been reported in MTX-treated osteosarcoma cells (60, 61). In fact, cell cycle progression is a key determinant of phospholipid metabolism and membrane homeostasis: G1/S blockade by RNAi during cellular growth stimulation decreases phosphatidylcholine levels, the main component of cell membranes (62). Coupling lipid metabolism with cell cycle progression is thought to be an evolutionary strategy to coordinate proliferation with cell and organelle growth. The nucleotide shortage provoked by MTX, with the subsequent halt of cell division in the S phase of the cycle, seems to trigger a signal to restrain phospholipid metabolism, thus preventing decompensation of cellular components.

MTX also decreased 2 bile acids, cholate and glycocholate. Bile acids are produced in the liver from cholesterol and might have been interiorized by the cells from the fetal serum present in the culture medium. MRP3, MRP8, and SLC19A1 (folate receptor) are membrane transporters capable of interiorizing MTX, cholate, and glycocholate (63, 64). Polymorphisms and expression levels of these transporters have been associated with prognosis in childhood ALL (65–68) due to impairment on chemotherapy internalization by leukemia cells. In our gene expression data, *SLC19A1* presented a negative correlation to MTX resistance, confirming that cellular sensitivity to the drug is related to enhanced drug uptake, whereas cholate and glycocholate concentrations were marginally associated with *SLC19A1* expression (*P* < 0.1) (data not shown). Bile acids such as taurocholate and cholate have proved to be efficient competitors of MTX absorption in hepatocytes (69, 70). Further investigation is required to determine whether seric bile acids could predict MTX uptake by primary leukemia cells and offer therapeutic guidance in clinics.

We found a significant inverse correlation between formate levels in either MTX-treated or untreated cells and cell doubling time or MTX resistance at 48 hours. Formate is a one-carbon molecule that can be synthesized and metabolized in folate-dependent or -independent reactions (71). Metabolic formate elimination appears to occur *via* its oxidation to carbon dioxide. Formate, THF, and ATP are combined to form 10-formyl-THF, which is then metabolized into THF, NADPH, and CO_2_. We found that formate levels increased upon MTX treatment (**Figure 1A**), possibly due to its accumulation by the lack of sufficient THF caused by the drug treatment, as formate production was shown to be markedly reduced in folate deficiency (72). The fact that we found formate levels in both untreated and treated cells to be negatively correlated with cell proliferation and MTX IC50 at 48 hours suggests that high formate level is a constitutive trait of MTX-sensitive cells. Further investigation will detail the mechanistic relationship between formate levels and MTX resistance, as well as show its applicability as an intracellular metabolic MTX marker.

Contrary to the conspicuous associations between formate and cell proliferation and MTX resistance, S-adenosylhomocysteine (SAH) levels were negatively correlated to these parameters only in MTX-treated cells. SAH is produced from methionine S-adenosylmethionine (SAM; the main methyl donor of the metabolism) demethylation and degraded to adenosine and L-homocysteine by the enzyme L-homocysteine hydrolase (AHCY). L-homocysteine is then converted to cystathionine (a precursor of glutathione) or is salvaged to methionine in a reaction that requires 5-methyl-THF (**Supplementary Figure 15**). We hypothesize that the lower levels of THF and its correlates (including 5-methyl-THF) in MTX-sensitive cells after drug treatment would contribute to SAH accumulation. Our findings showed that methionine cycle intermediates in MTX-treated cells correlate to antifolate resistance, suggesting their potential use as a metabolic biomarker of MTX response that deserves further investigation.

Asparagine regulates mTORC1 activity (73) and can act as an exchange factor, influencing serine metabolism and nucleotide synthesis by regulating serine uptake (74). We speculate that constitutive higher levels of asparagine would predispose cells to resist MTX insult by conferring an extra pool of molecules to be used as exchange factors for serine uptake, leading to 5,10-methylene-THF production and, ultimately, to pyrimidine biosynthesis even in an MTX-mediated DHFR inhibition scenario.

Guanosine is a purine nucleoside and indirect precursor of GTP; it is plausible to assume that higher constitutive levels of this intermediate would confer a natural resistance to MTX, although, to the best of our knowledge, these findings (ie, a positive correlation between basal asparagine and guanosine levels and MTX resistance) have never been described in the literature.

MTX’s ability to induce the formation of ROS has already been described extensively in the literature (75–77). Our work showed that GSH levels were positively associated with MTX resistance in both untreated and treated cells, meaning that cell’s constitutive antioxidant defense is predictive of MTX resistance and remains stable even after drug insult. We found the expression of 8 genes coding for enzymes involved in the GSH metabolism to be positively associated with the metabolite’s level. Among these are the genes *GSTM3* and *GSTA4*, which code for GSH S-transferases capable of MTX glutathionylation (78). Intriguingly, *GSTM3* expression in ALL lymphoblasts was positively associated with favorable prognosis (79). Some GSH hydrolases (or gamma-glutamyltransferases [GGTs]) were also correlated with GSH levels. GGTs participate in the gamma-glutamyl cycle, a route of synthesis/degradation of GSH and detoxification of drugs and xenobiotics (80), which explains the positive association we observed between *GGT1* expression with both GSH content and MTX resistance. *GGT’s* expression decrease during ALL treatment returns to normal levels during the chemotherapy-induced remission stage (81), though circulating enzyme levels did not show prognostic value (82).

Parallel to the GSH detox metabolism, thioredoxins are small oxidoreductase enzymes with a dithiol disulfide site that are essential for mammalian life and involved in cellular redox balance and signaling (83). When thioredoxins are oxidized, thioredoxin reductases (TrxR’s) convert them back to their reduced state at the expense of NADPH, which is oxidized to NADP+. Interestingly, among the 3 thioredoxin reductase isozymes found in mammals, only thioredoxin reductase 3 (TXNRD3) contains an additional N-terminal glutaredoxin domain, which makes it a participant of both thioredoxin and glutathione systems. In this study, we found *TXNRD3* expression to be positively associated with both GSH content and MTX resistance. TrxR’s enzymes are overexpressed or constitutively expressed in various cancers, contributing to tumor growth and chemoresistance (84), so its inhibition has drawn increasing attention. There are several studies demonstrating that pharmacological inhibitors of TrxR’s can be effective as single agents or combined with chemo/radiotherapy in a variety of cancer types (85, 86). In a study of 14 cancer cell lines from diverse tissues treated with a panel of 19 chemotherapeutic agents, MTX resistance was directly related to the enzymatic activity of glutathione reductase and thioredoxin reductase but not to the intracellular concentration of GSH, which, in turn, was associated with ThioTEPA (an alkylating agent) and doxorubicin resistance (87). Recently, by using CRISPR-Cas9, Oshima et al (88) showed that *TXNRD3* (and *TXNRD2*) knockout in REH cells resulted in increased sensitivity to MTX, confirming the role of the thioredoxin system on MTX detoxification.

In this study, the GSH synthesis promoter N-acetylcysteine was shown to increase MTX resistance and proliferation of ALL cell lines. NAC’s protective effect against diverse drugs in varied cancer types has been extensively described in the literature. However, we noticed an inverse correlation between GSH levels and NAC effect, a phenomenon that we attribute to the feedback loop that coordinates GSH synthesis, through which GSH inhibits GCL activity (89) and thus decreases metabolite synthesis when its cellular pool is already full.

If, by one hand, NAC supplementation boosts MTX resistance in ALL cell lines, co-treatment with GSH scavengers had a marginal effect on sensitizing cells to the antifolate. We hypothesized that this was due to the thioredoxin metabolism, the other antioxidant system of the cell which can compensate an impaired or blocked GSH metabolism (90, 91). We demonstrated the interplay between both antioxidant systems by treating ALL cells with auranofin, a thioredoxin reductase, and BSO, a GSH synthesis inhibitors. The remarkable combination effect observed has been previously reported in head and neck cancer (92, 93), breast cancer (94), and lymphoma and multiple myeloma (95) cells. In some cases, the thioredoxin system may be even more important than GSH to protect cells from oxidative stress. For instance, in prostate and breast cancer, the synergistic combination of 2-deoxy-glucose (2DG) with DHEA (a phosphate-pentose pathway inhibitor) was potentiated by auranofin but not BSO, whereas the cytotoxic effect was completely abrogated by the addition of NAC (96). Our metabolomic results suggest that MTX also inhibits the pentose-phosphate pathway — a theoretically similar effect to that caused by DHEA. Therefore, like the duo MTX+DHEA, the MTX+BSO+auranofin triple combination may deserve further investigation in primary ALL.

Studies in fibroblasts showing that folic acid supplementation abrogates the cytotoxic effects of arsenite have led to the suggestion that MTX could synergize with ATO (97). By causing a relative methyl insufficiency, MTX could potentiate the effects of ATO in the presence of excess folate. We investigated the effects of the thioredoxin system on MTX resistance *in vitro* by treating ALL cell lines with thioredoxin reductase inhibitors in the presence of the antifolate. Both auranofin and ATO — a rheumatologic and a chemotherapy agent, respectively — potentiated MTX cytotoxicity slightly in a few cell lines, indicating that the thioredoxin antioxidant system could be protecting the cells from the oxidative stress caused by MTX.

ATO has been used against acute promyelocytic leukemia (APL) for the last 2 decades (98), usually in combination with all-*trans* retinoic acid (ATRA) (99, 100) during both induction and consolidation phases. Prolonged maintenance with continuous low-dose chemotherapy (which includes MTX) and intermittent ATRA was beneficial for high-risk APL (101–103). Aligned with this fact and our *in vitro* data, we showed that ATO potentiated MTX cytotoxicity in an *in vivo* model of an aggressive primary murine ALL and in 1 of 4 human ALL PDXs tested, for which tumor burden in the peripheral blood was significantly decreased by the drug combination as well. Interestingly, this ATO-responsive PDX presented a particular MTX-resistant phenotype, whereas in the other 3 cases, single-agent MTX was sufficient to control leukemia progression, thus hampering any possible additive effect by ATO. Both high-dose MTX and ATO are known to cause gastrointestinal toxicity (104, 105), and although no mouse that received either MTX or ATO single-agent experienced severe gastrointestinal toxicity, this was so for 5 out of 24 animals treated with ATO+MTX, indicating that future studies are required to better evaluate the safety of the ATO+MTX combination for the treatment of ALL patients.

Although relapse/refractory ALL did not respond to single-agent ATO in a previous clinical trial (106), which is in line with our PDX results, few studies have investigated its benefits in ALL in combination with other drugs. For instance, Szymanska et al (44) tested the addition of ATO (in the same dose and regimen as ours) with vincristine, dexamethasone, and asparaginase (VXL) in 4 ALL PDXs. Although ATO, in combination with VXL, statistically improved the progression delay of 2 xenografts tested, the authors concluded that the benefit appears unlikely to be of biological significance. We believe that our study brings a new perspective by proposing the use of thioredoxin reductase inhibitors, specially ATO, in combination with MTX. Further investigation should aim to test the Auranofin+MTX combination, the benefits of a third drug inclusion (such as BSO), and the molecular features of the ALL that would most benefit from this new drug combination.

MTX’s mechanisms of action and resistance are still being rewritten even after several decades of research and therapeutic use. Our study showed that the antioxidant systems of the cell are an important component of leukemia resistance to MTX, and targeting these pathways, especially the thioredoxin antioxidant system, may contribute to re-sensitize ALL to MTX. In a broad sense, this work illustrates how metabolomics can be employed as an alternative approach to unravel unexplored mechanisms of action and/or resistance of drugs, leveraging insights about new drug combinations with translational potential for life-threatening diseases such as cancer.

## Data availability statement

The data presented in the study are deposited in the Gene Expression Omnibus (GEO) repository, accession number GSE218348.

## Ethics statement

The studies involving human participants were reviewed and approved by Comitê de Ética em Pesquisa, Centro Infantil Boldrini, CAAE 34601120.7.0000.5376. Written informed consent to participate in this study was provided by the participants’ legal guardian/next of kin. The animal study was reviewed and approved by Comissão de Ética no Uso de Animais (CEUA), Instituto de Biologia, Universidade Estadual de Campinas (UNICAMP), protocol 4557-1(A)/2018; and Comissão de Ética no Uso de Animais (CEUA), Centro Infantil Boldrini, protocol 0016-2020.

## Author contributions

RRC, CPSM, SRB, ACMZ and JAY conceived the study. RRC, CPSM, NMR, LLA, JRC, YTL, SSM, and PRS performed the experiments and analyzed the data. ACMZ, SRB and JAY supervised the study. RC and JY wrote the manuscript. All authors contributed to the article and approved the submitted version.
