# Erratum: Glutathione levels are associated with methotrexate resistance in acute lymphoblastic leukemia cell lines

**DOI:** 10.3389/fonc.2023.1190120

**Published:** 2023-03-28

**Authors:** Frontiers Production Office

**Affiliations:** Frontiers Media SA, Lausanne, Switzerland

**Keywords:** acute lymphoblastic leukemia, methotrexate, glutathione, metabolomics, drug resistance, arsenic trioxide, thioredoxin reductase

Due to a production error, the given names, and surnames of the authors in the citation were changed to “**Renatino Canevarolo R, Pereira de Souza Melo C, Moreno Cury N, Luiz Artico LL, Ronchi Corrêa J, Tonhasca Lau Y, Sousa Mariano S, Reddy Sudalagunta P, Regina Brandalise S, Carolina de Mattos Zeri A and Andrés Yunes J (2022) Glutathione levels are associated with methotrexate resistance in acute lymphoblastic leukemia cell lines. Front. Oncol. 12:1032336. doi: 10.3389/fonc.2022.1032336**”. The correct citation is “**Canevarolo RR, Melo CPS, Cury NM, Artic LL, Corrêa JR, Lau YT, Mariano SS, Sudalagunta PR, Brandalise SR, Zeri ACM, and Yunes JA (2022) Glutathione levels are associated with methotrexate resistance in acute lymphoblastic leukemia cell lines. Front. Oncol. 12:1032336. doi: 10.3389/fonc.2022.1032336**”. The publisher apologizes for this mistake.

The original version of this article has been updated.

