# Supplementary material for: Glutathione levels are associated with methotrexate resistance in acute lymphoblastic leukemia cell lines
Source: Front Oncol. 2022 Dec 1;12:1032336. doi: 10.3389/fonc.2022.1032336 (PMC9751399; doi:10.3389/fonc.2022.1032336)
Supplement: Supplementary file 1 [file DataSheet_1.pdf]

a

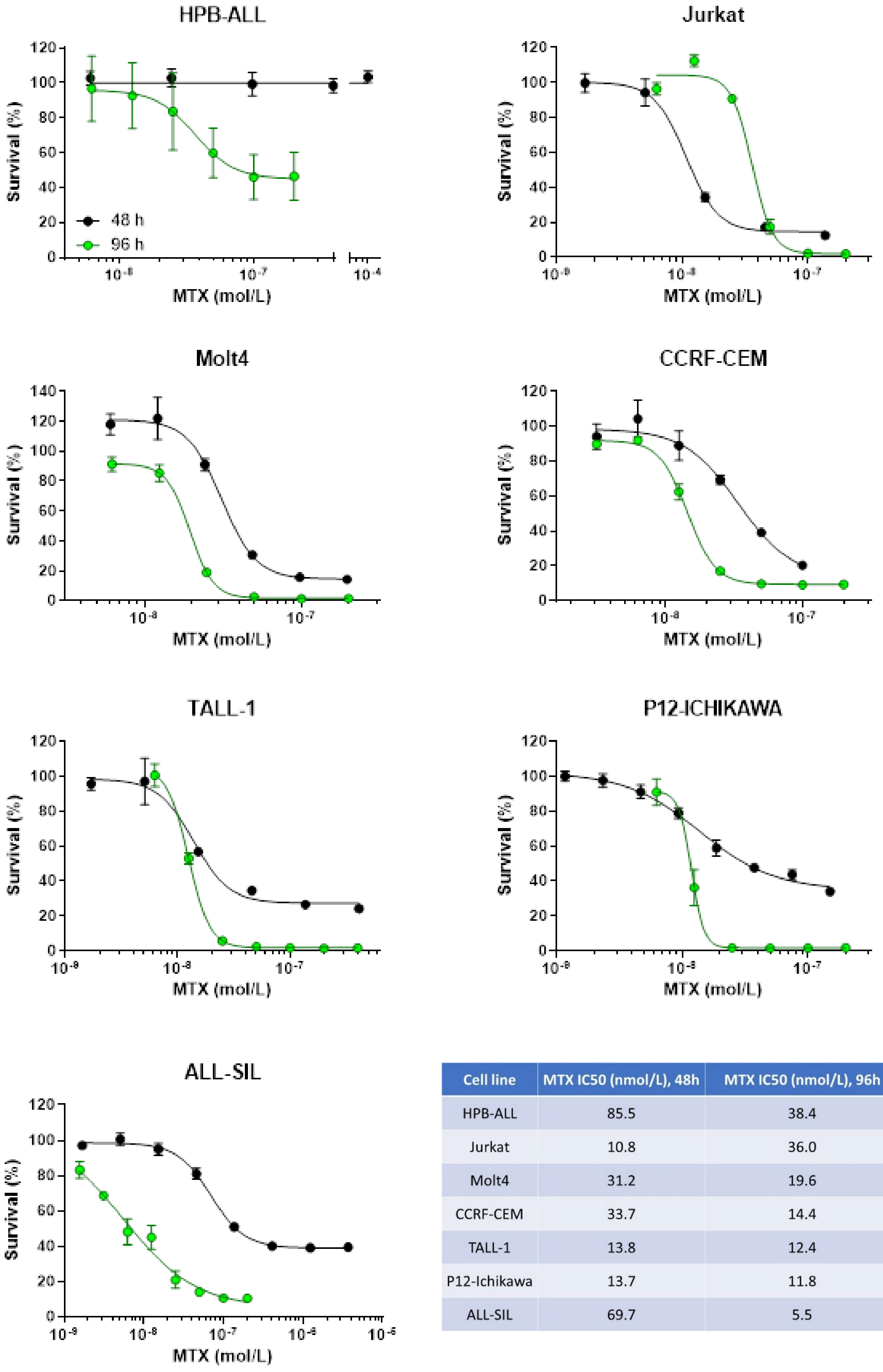

Cell line	MTX IC50 (nmol/L), 48h	MTX IC50 (nmol/L), 96h
HPB-ALL	85.5	38.4
Jurkat	10.8	36.0
Molt4	31.2	19.6
CCRF-CEM	33.7	14.4
TALL-1	13.8	12.4
P12-Ichikawa	13.7	11.8
ALL-SIL	69.7	5.5

b

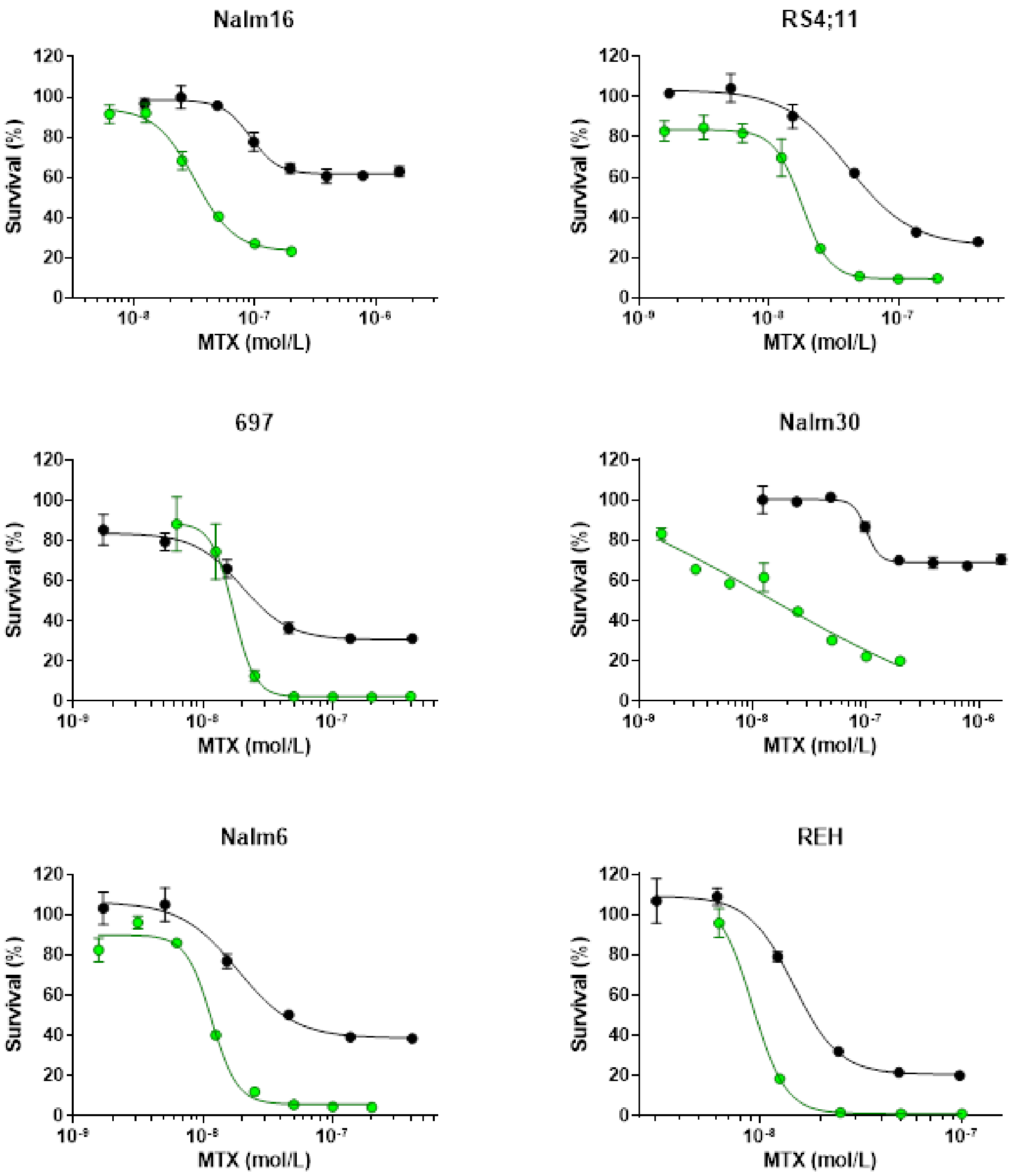

Cell line	MTX IC50 (nmol/L), 48h	MTX IC50 (nmol/L), 96h
Nalm16	91.8	31.7
RS4;11	41.1	17.9
697	19.9	17.0
Nalm30	101.0	14.2
Nalm6	18.5	11.6
REH	14.7	9.1

Supplementary Figure 1. MTX dose-response curves of **(a)** T-ALL and **(b)** BCP-ALL cell lines. Survival at each dose was determined in relation to negative controls (defined as 100%). Doses were tested in triplicates. Black: 48 h of treatment; green: 96 h. The legend brings the IC50 values for each curve. MTT reduction assay (similar results were obtained with calcein AM).

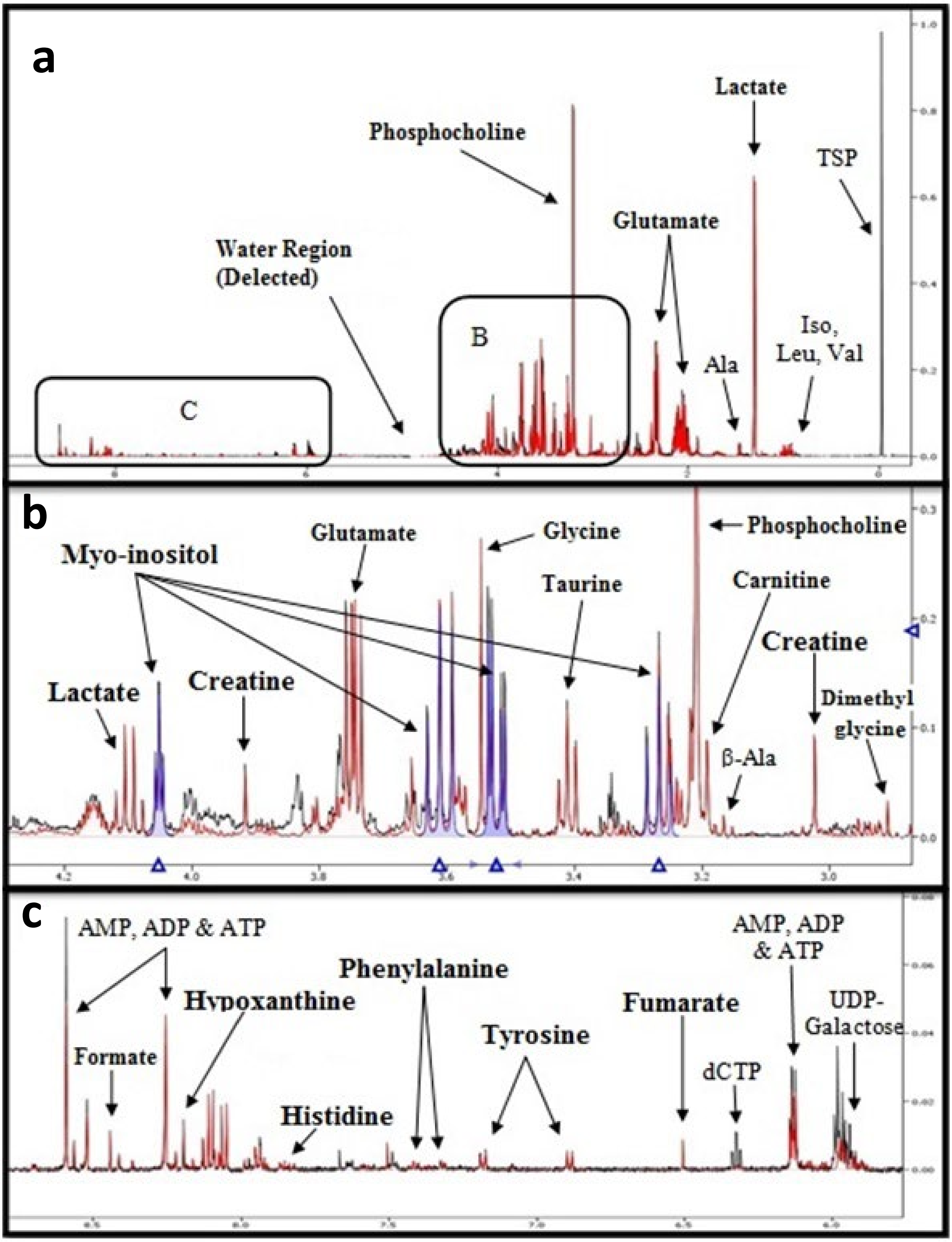

Supplementary Figure 2. **(a)** ^{1}H-NMR representative spectrum; **(b)** and **(c)** details some compounds found at their respective spectral region, as indicated in **(a)**.

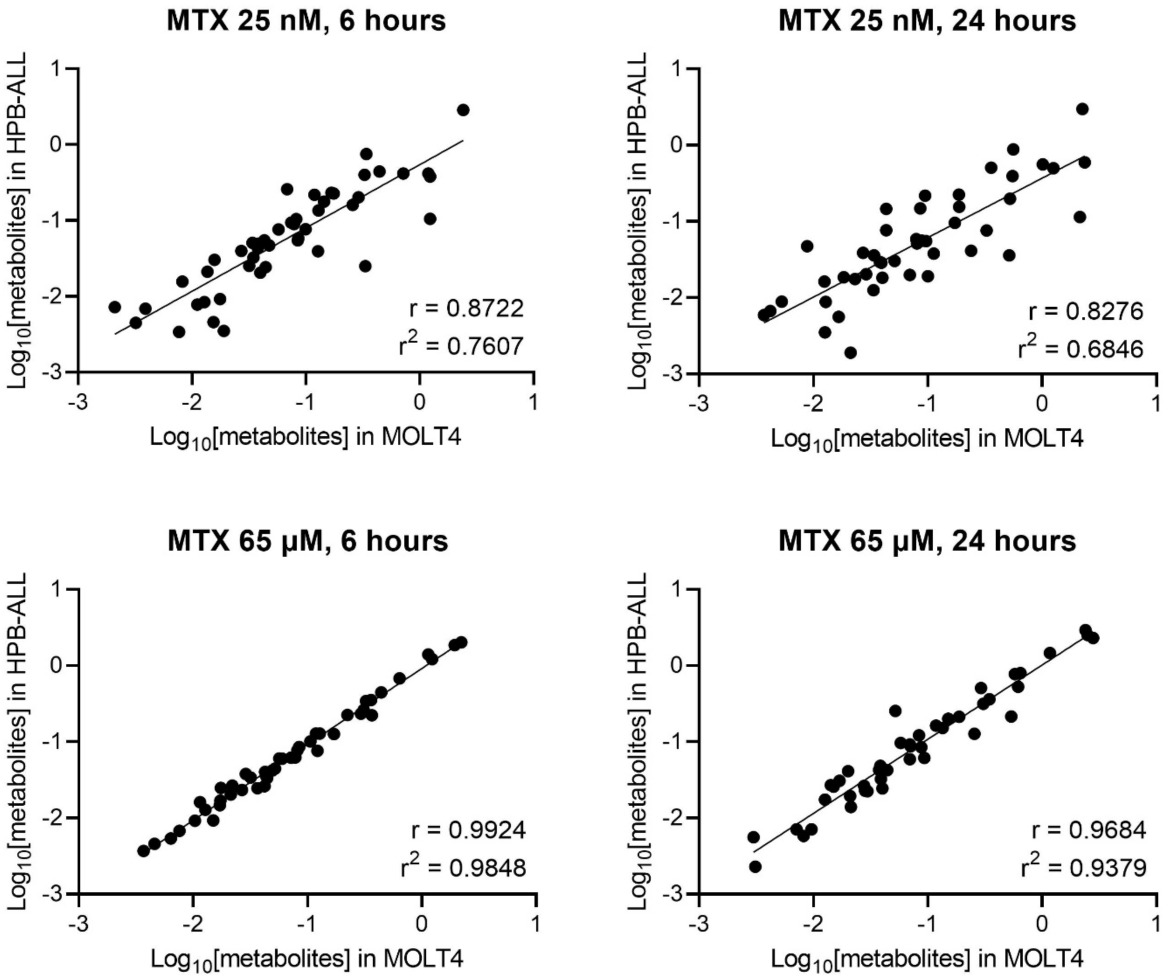

Supplementary Figure 3. Correlation between metabolites concentrations found in MTX-resistant HPB-ALL and MTX-sensitive MOLT4 under different treatment conditions. The greatest variation was found in MTX 25 nM at 24 hours.

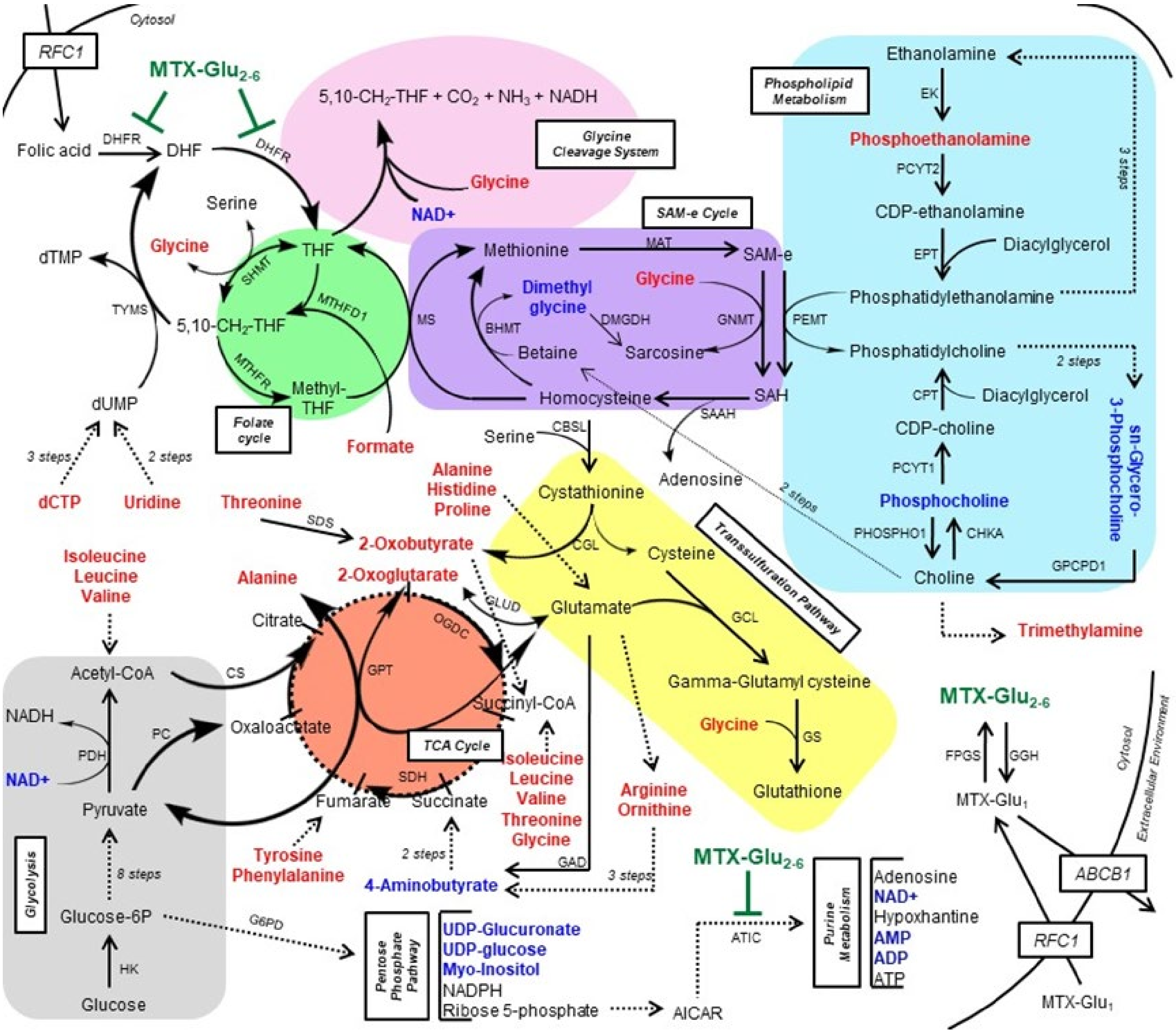

Supplementary Figure 4. Scheme congregating all measured MTX-modulated metabolites. Metabolites augmented or decreased after drug treatment are depicted in red and blue, respectively. MTX-Glu$_{2-6}$: the intracellular polyglutamylated form of MTX. Green traces represent the inhibitory effect of MTX-Glu$_{2-6}$ on its main target enzymes, DHFR and ATIC.

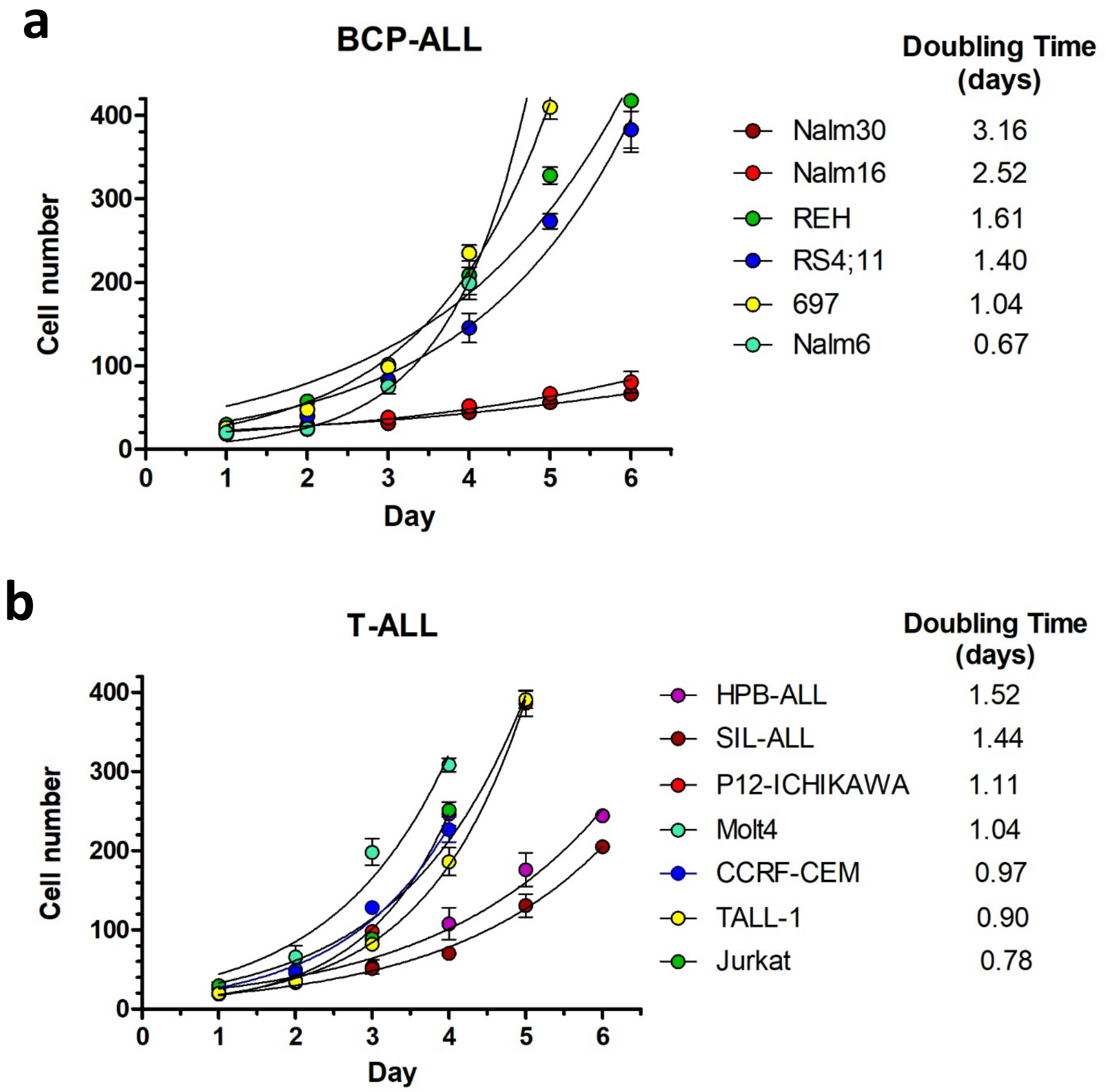

Supplementary Figure 5. (a) BCP-ALL and **(b)** T-ALL cell lines proliferation curves. Cells were allowed to divide in culture medium over six days in order to have their doubling time determined.

a

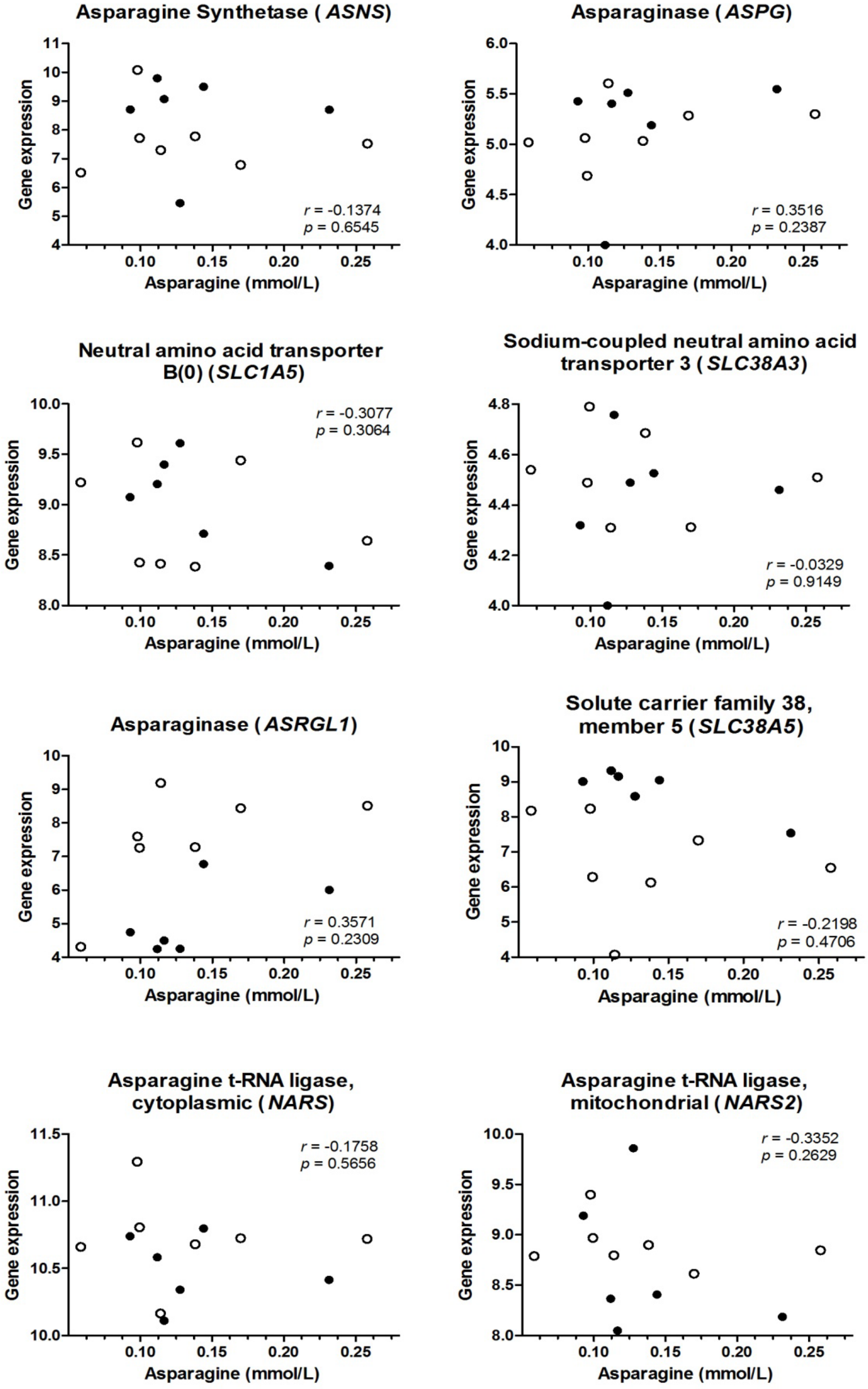
Asparagine Synthetase (ASNS)
r = -0.1374
p = 0.6545
Asparaginase (ASPG)
r = 0.3516
p = 0.2387
Neutral amino acid transporter B(0) (SLC1A5)
r = -0.3077
p = 0.3064
Sodium-coupled neutral amino acid transporter 3 (SLC38A3)
r = -0.0329
p = 0.9149
Asparaginase (ASRGL1)
r = 0.3571
p = 0.2309
Solute carrier family 38, member 5 (SLC38A5)
r = -0.2198
p = 0.4706
Asparagine t-RNA ligase, cytoplasmic (NARS)
r = -0.1758
p = 0.5656
Asparagine t-RNA ligase, mitochondrial (NARS2)
r = -0.3352
p = 0.2629
Gene expression
Asparagine (mmol/L)

b

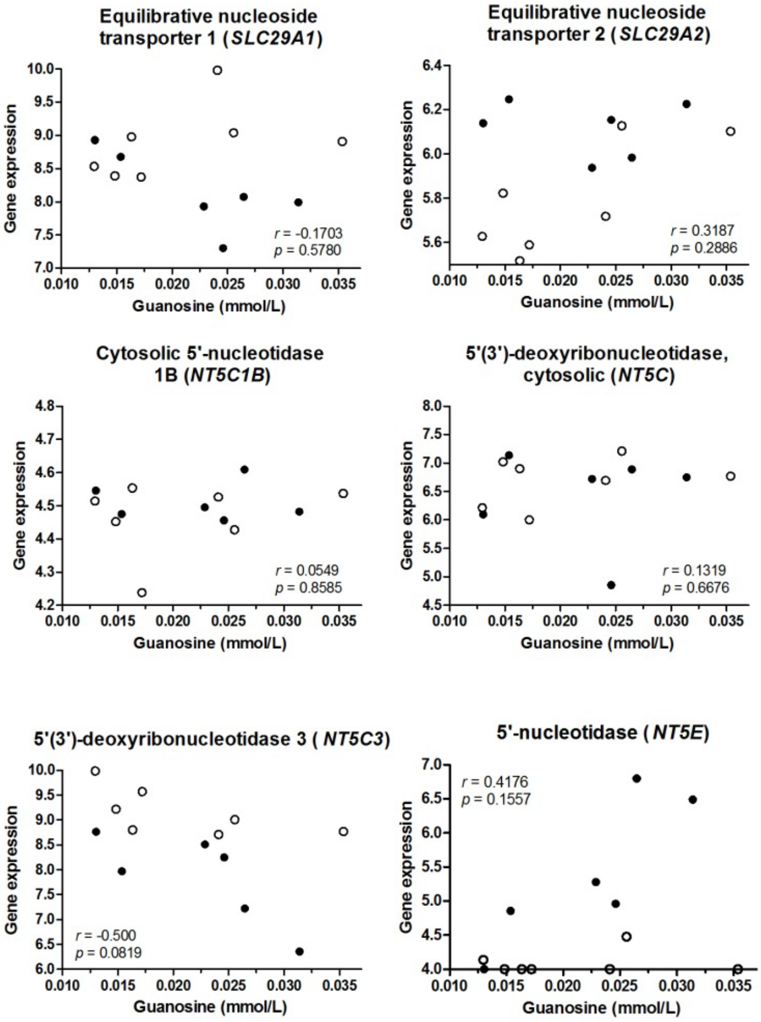
Equilibrative nucleoside transporter 1 (SLC29A1)
r = -0.1703
p = 0.5780
Equilibrative nucleoside transporter 2 (SLC29A2)
r = 0.3187
p = 0.2886
Cytosolic 5'-nucleotidase 1A (NT5C1A)
r = 0.0494
p = 0.8725
Cytosolic 5'-nucleotidase 1B (NT5C1B)
r = 0.0549
p = 0.8585
5'(3')-deoxyribonucleotidase, cytosolic (NT5C)
r = 0.1319
p = 0.6676
Cytosolic purine 5'-nucleotidase (NT5C2)
r = -0.3462
p = 0.2466
5'(3')-deoxyribonucleotidase 3 (NT5C3)
r = -0.500
p = 0.0819
5'-nucleotidase (NT5E)
r = 0.4176
p = 0.1557
5'(3')-deoxyribonucleotidase, mitochondrial (NT5M)
r = 0.0714
p = 0.8166
Gene expression
Guanosine (mmol/L)

c

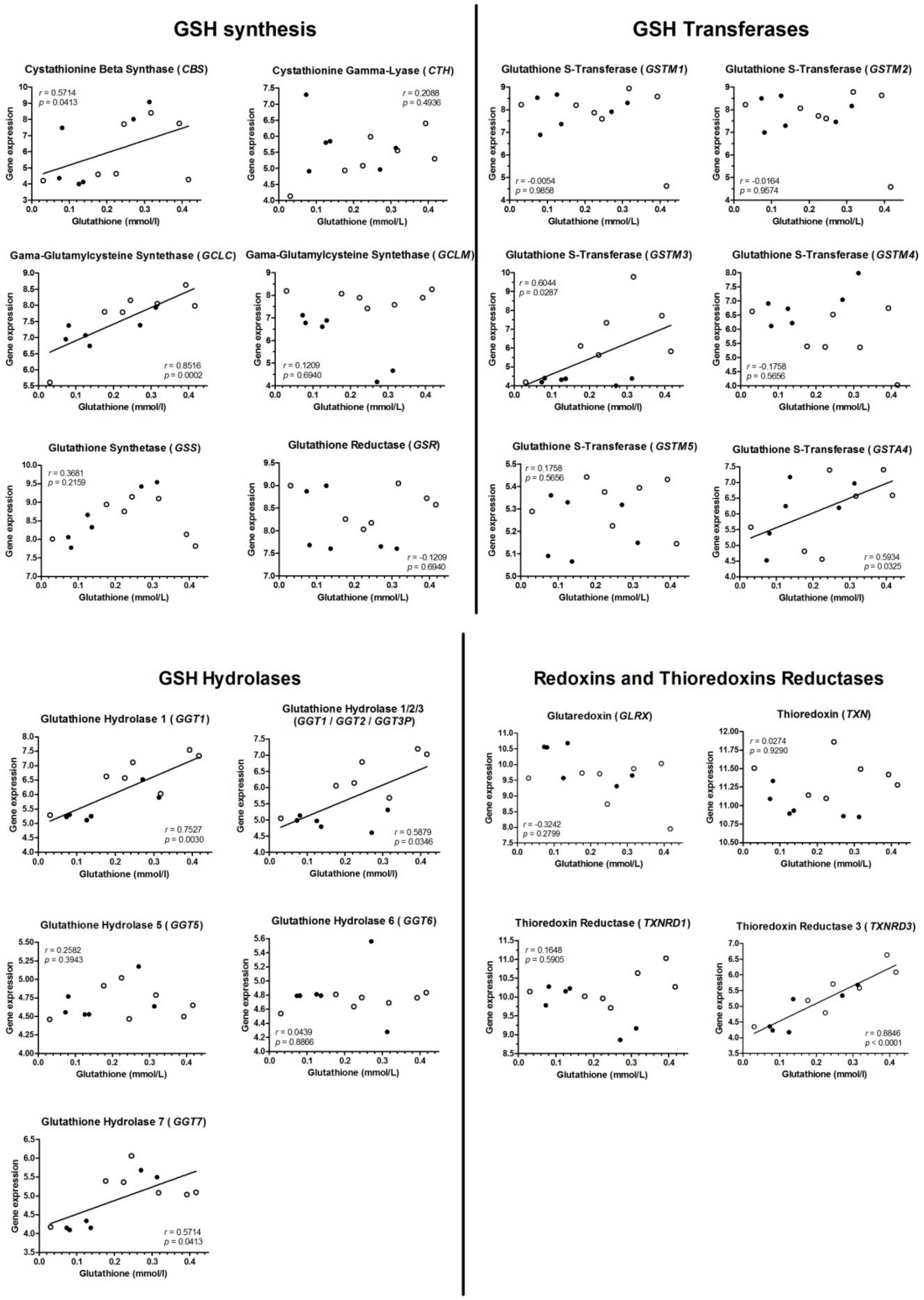
GSH synthesis
Cystathionine Beta Synthase (CBS)
r = 0.5714
p = 0.0413
Cystathionine Gamma-Lyase (CTH)
r = 0.2088
p = 0.4936
Gama-Glutamylcysteine Syntethase (GCLC)
r = 0.8516
p = 0.0002
Gama-Glutamylcysteine Syntethase (GCLM)
r = 0.1209
p = 0.6940
Glutathione Synthetase (GSS)
r = 0.3681
p = 0.2159
Glutathione Reductase (GSR)
r = -0.1209
p = 0.6940
GSH Transferases
Glutathione S-Transferase (GSTM1)
r = -0.0054
p = 0.9858
Glutathione S-Transferase (GSTM2)
r = -0.0164
p = 0.9574
Glutathione S-Transferase (GSTM3)
r = 0.6044
p = 0.0287
Glutathione S-Transferase (GSTM4)
r = -0.1758
p = 0.5656
Glutathione S-Transferase (GSTM5)
r = 0.1758
p = 0.5656
Glutathione S-Transferase (GSTA4)
r = 0.5934
p = 0.0325
GSH Hydrolases
Glutathione Hydrolase 1 (GGT1)
r = 0.7527
p = 0.0030
Glutathione Hydrolase 1/2/3 (GGT1 / GGT2 / GGT3P)
r = 0.5879
p = 0.0346
Glutathione Hydrolase 5 (GGT5)
r = 0.2582
p = 0.3943
Glutathione Hydrolase 6 (GGT6)
r = 0.0439
p = 0.8866
Glutathione Hydrolase 7 (GGT7)
r = 0.5714
p = 0.0413
Redoxins and Thioredoxins Reductases
Glutaredoxin (GLRX)
r = -0.3242
p = 0.2799
Thioredoxin (TXN)
r = 0.0274
p = 0.9290
Thioredoxin Reductase (TXNRD1)
r = 0.1648
p = 0.5905
Thioredoxin Reductase 3 (TXNRD3)
r = 0.8846
p < 0.0001
Gene expression
Glutathione (mmol/L)

c (cont.)

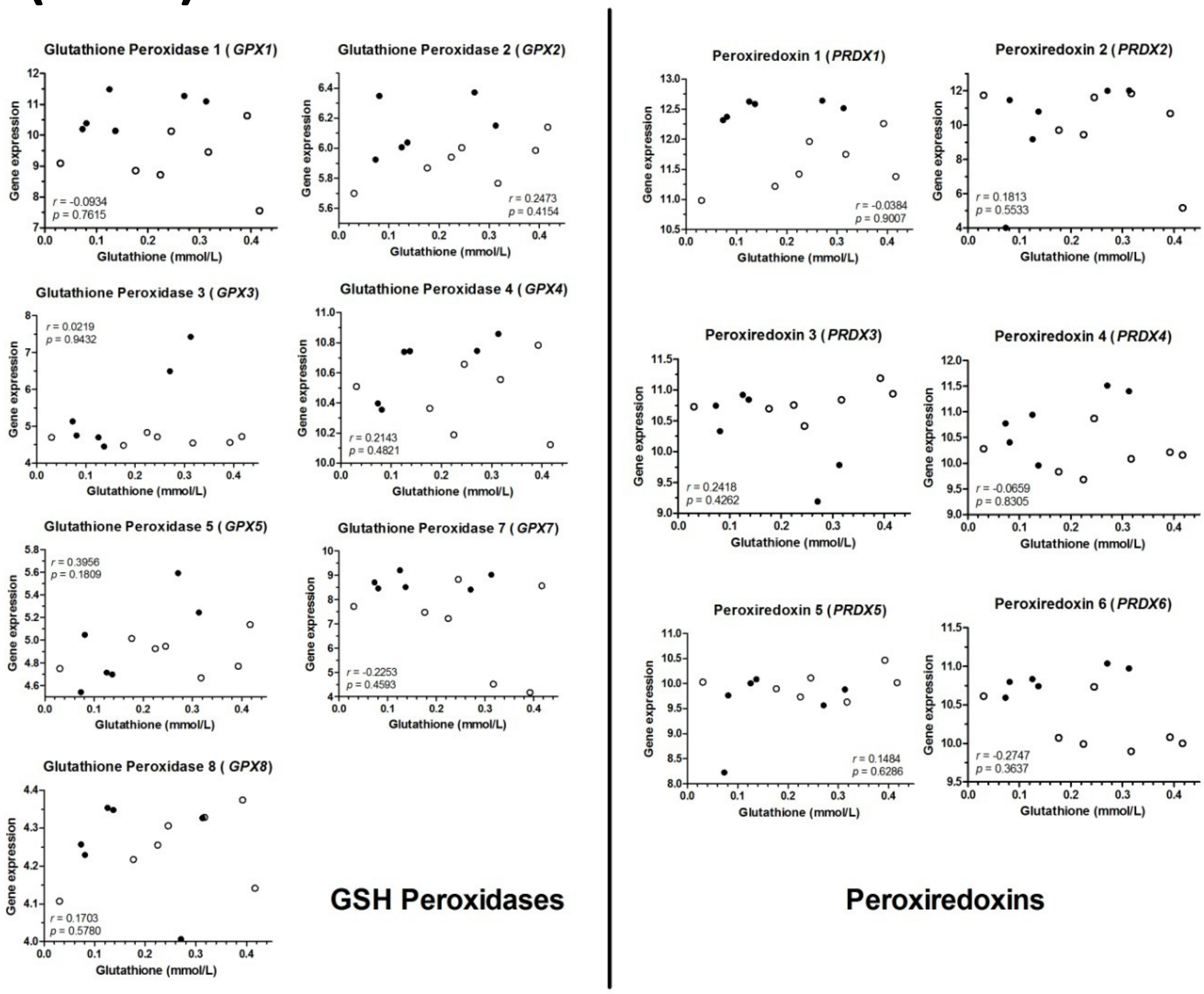

Supplementary Figure 6. Correlation between **(a)** asparagine, **(b)** guanosine and **(c)** glutathione levels and the expression of genes involved in their respective metabolisms. r = Spearman correlation coefficient. *p* = significance level. Black dots: BCP-ALL cell lines; white dots: T-ALL cell lines.

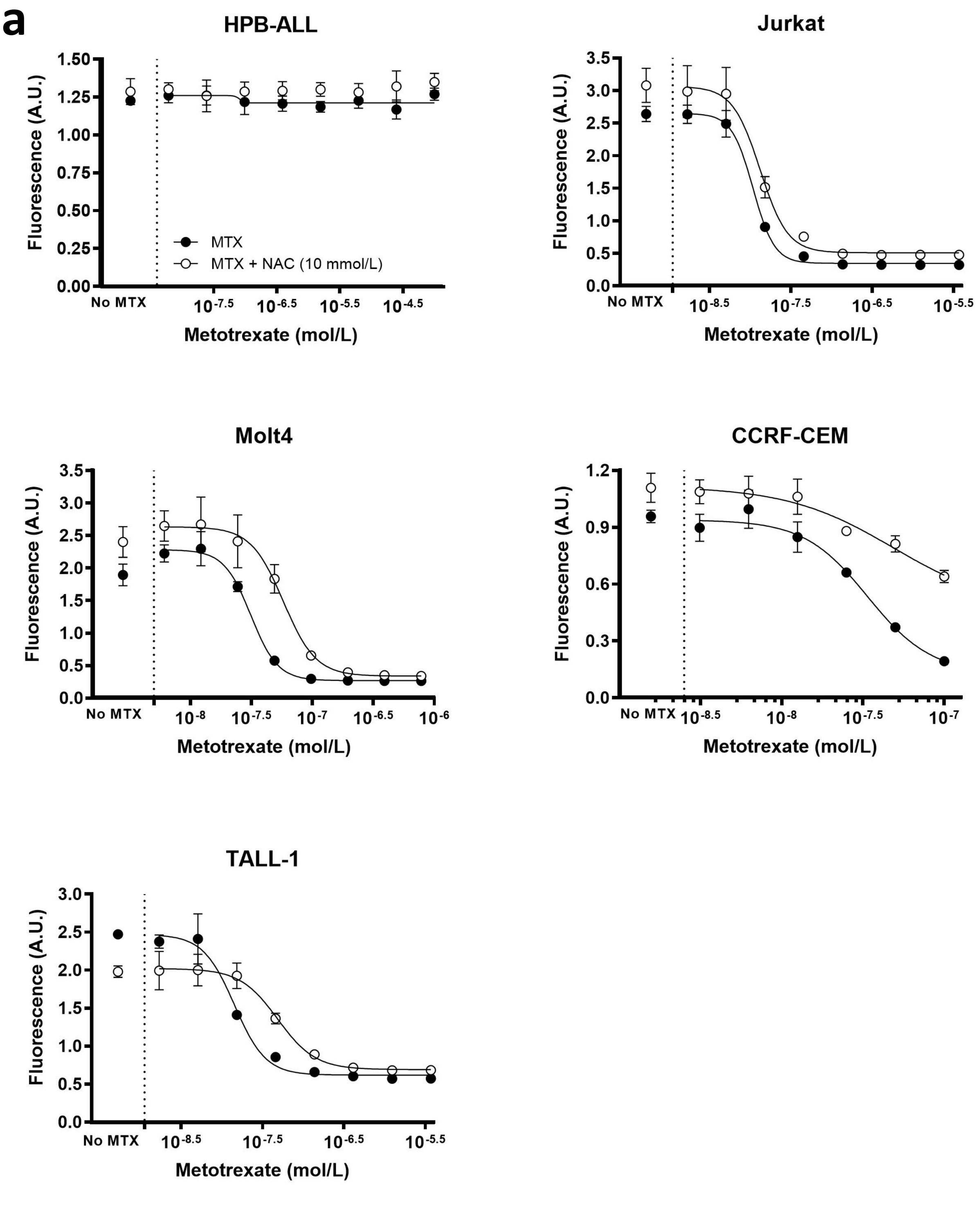
a
HPB-ALL
Jurkat
Molt4
CCRF-CEM
TALL-1
Fluorescence (A.U.)
Metotrexate (mol/L)
No MTX
MTX
MTX + NAC (10 mmol/L)

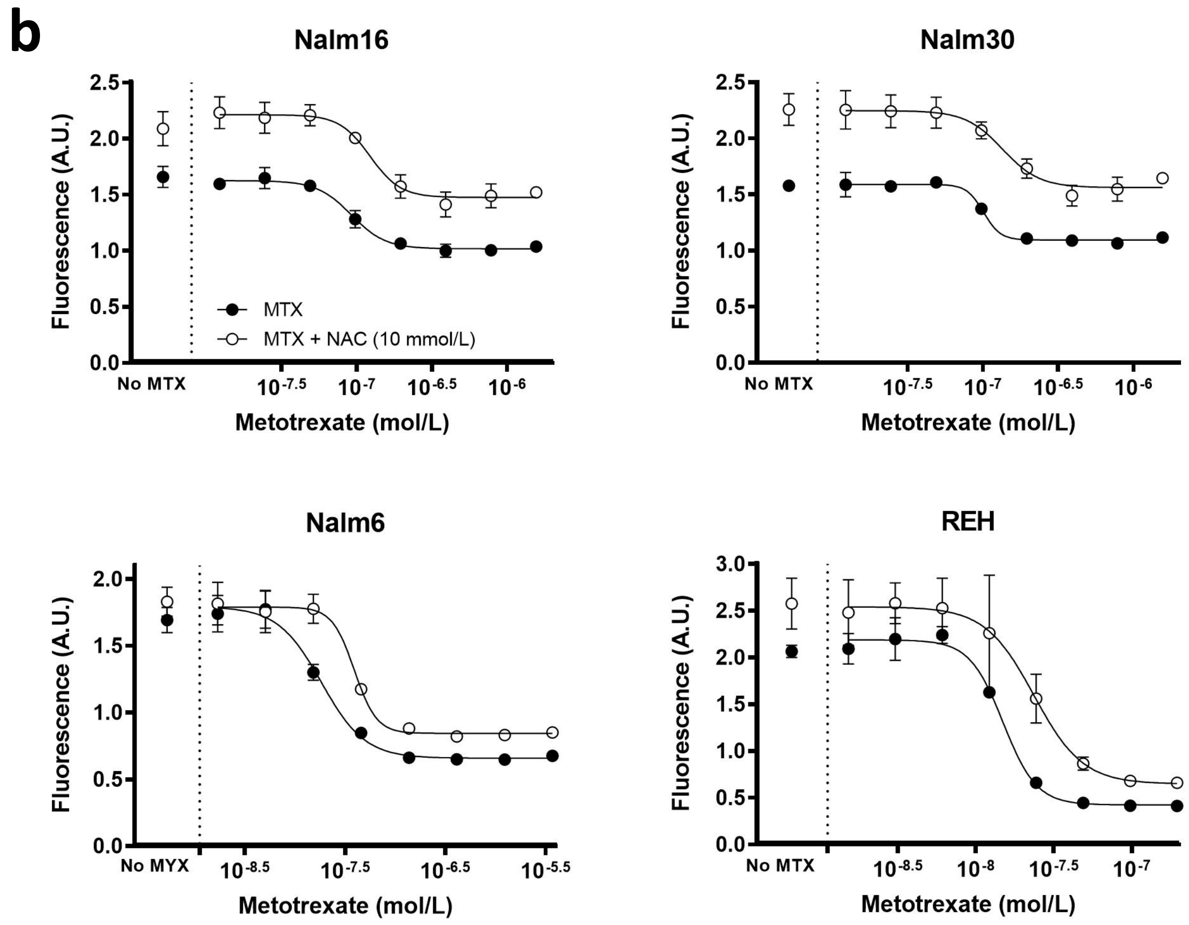

Supplementary Figure 7. Dose-response curves of **(a)** T-ALL and **(b)** BCP-ALL cell lines to MTX as a single agent (black beads) or in combination with N-acetylcysteine (NAC, 10 mmol/l, white beads). Error bars represent standard deviation from three biological replicates. A.U. = arbitrary units.

a

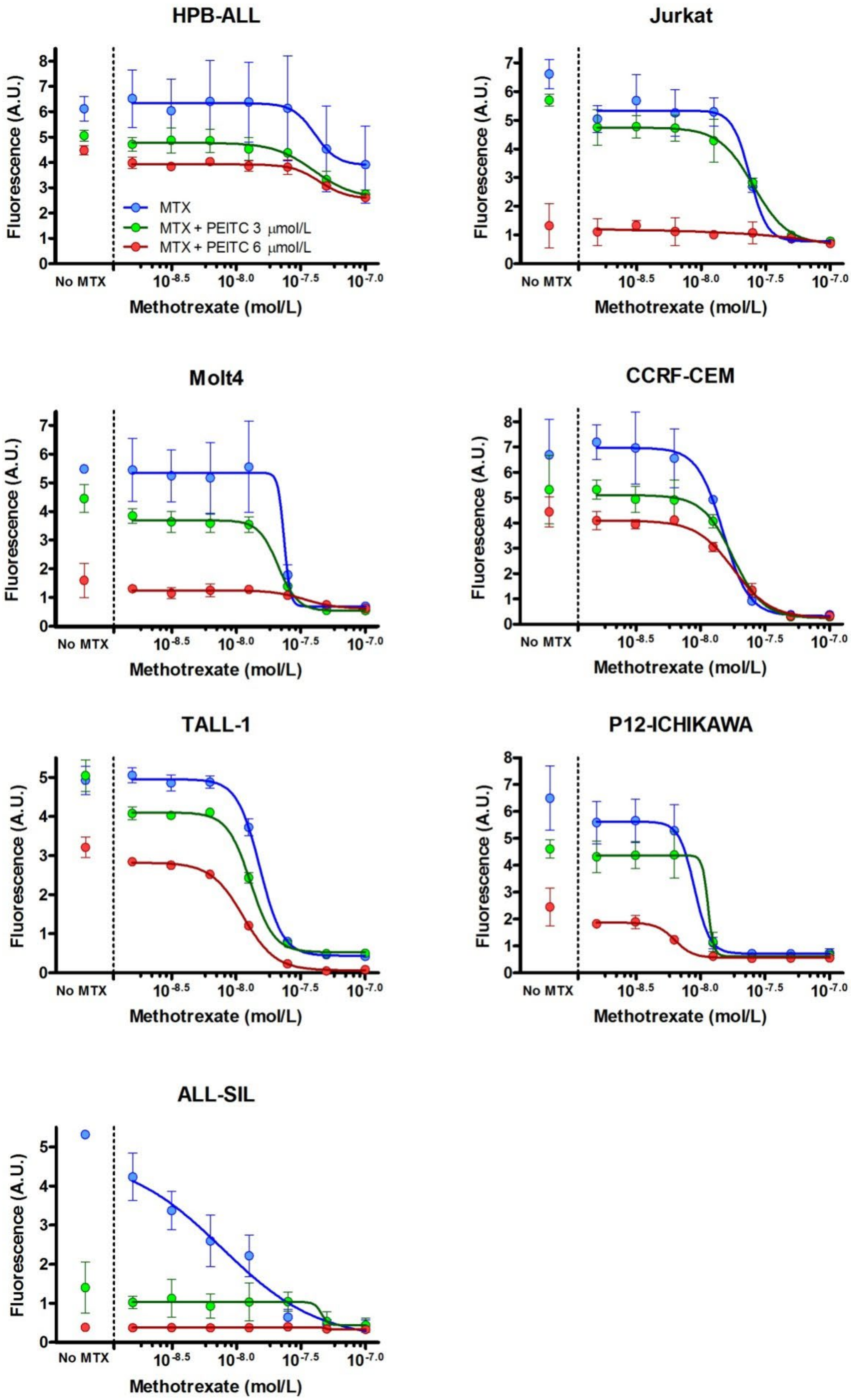
HPB-ALL
Jurkat
Molt4
CCRF-CEM
TALL-1
P12-ICHIKAWA
ALL-SIL
Fluorescence (A.U.)
No MTX
$10^{-8.5}$
$10^{-8.0}$
$10^{-7.5}$
$10^{-7.0}$
Methotrexate (mol/L)
MTX
MTX + PEITC 3 μmol/L
MTX + PEITC 6 μmol/L

b

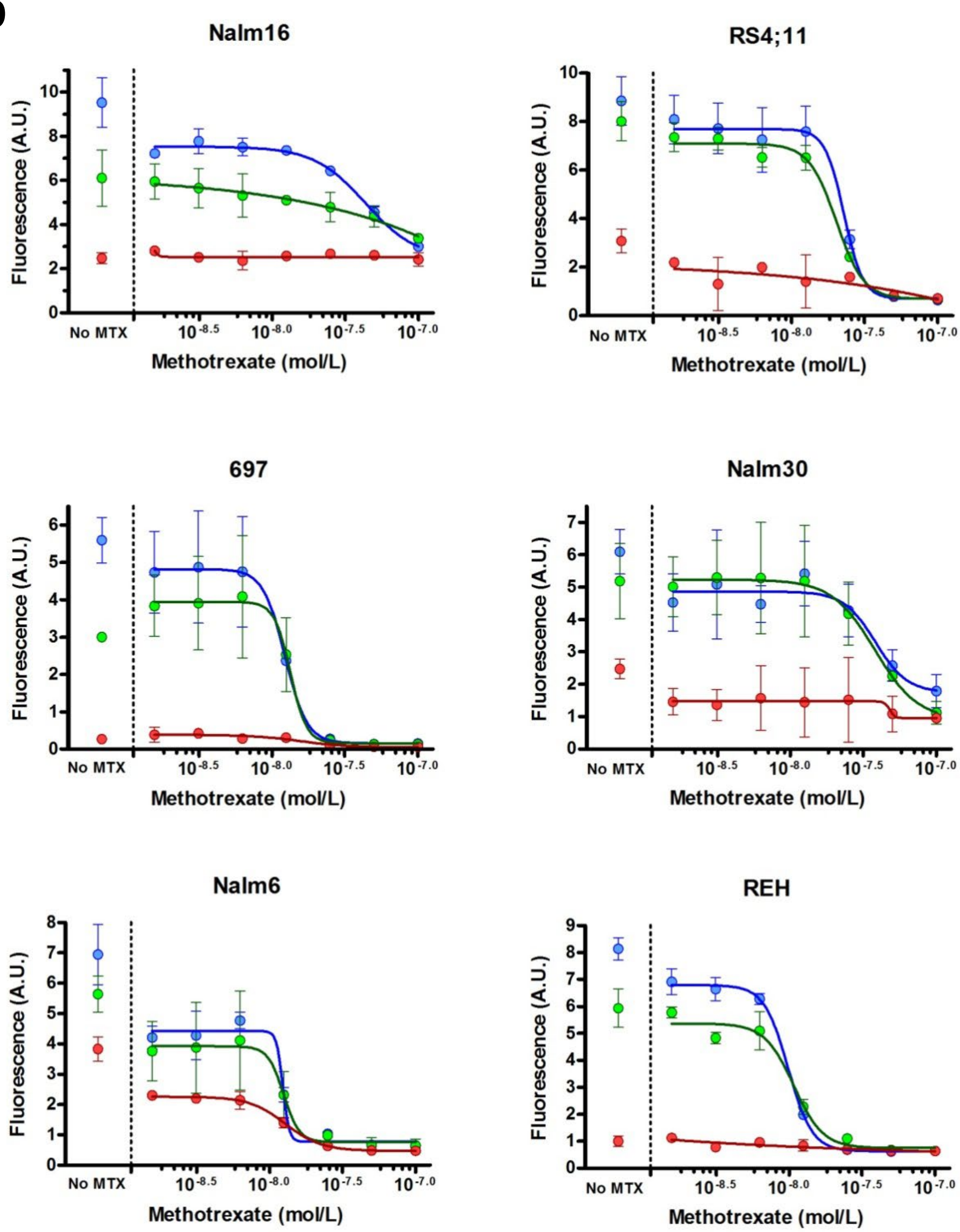

Nalm16
RS4;11
697
Nalm30
Nalm6
REH
Fluorescence (A.U.)
No MTX
$10^{-8.5}$
$10^{-8.0}$
$10^{-7.5}$
$10^{-7.0}$
Methotrexate (mol/L)

c

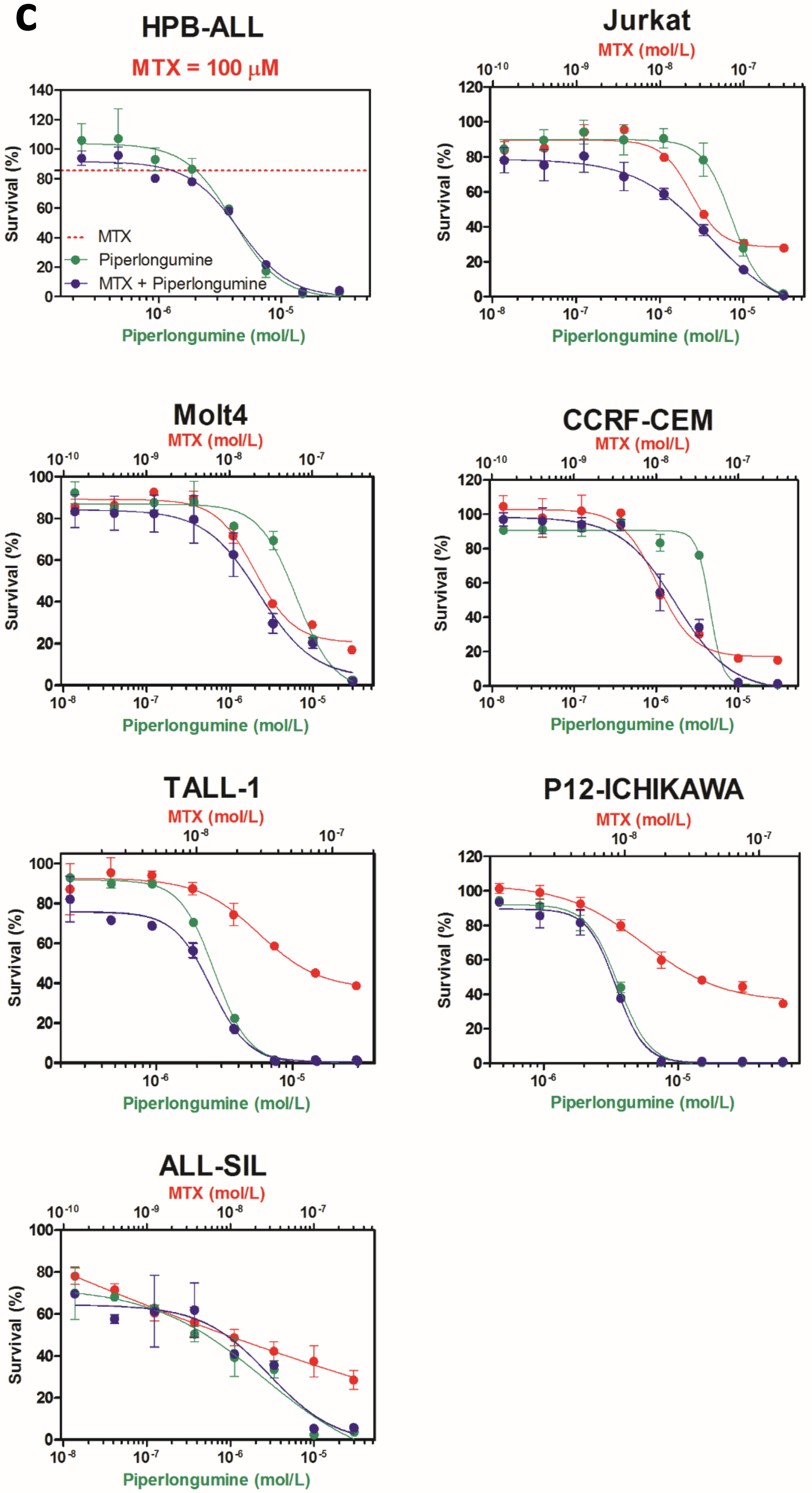
HPB-ALL
MTX = 100 μM
Survival (%)
MTX
Piperlongumine
MTX + Piperlongumine
Piperlongumine (mol/L)
Jurkat
MTX (mol/L)
Survival (%)
Piperlongumine (mol/L)
Molt4
MTX (mol/L)
Survival (%)
Piperlongumine (mol/L)
CCRF-CEM
MTX (mol/L)
Survival (%)
Piperlongumine (mol/L)
TALL-1
MTX (mol/L)
Survival (%)
Piperlongumine (mol/L)
P12-ICHIKAWA
MTX (mol/L)
Survival (%)
Piperlongumine (mol/L)
ALL-SIL
MTX (mol/L)
Survival (%)
Piperlongumine (mol/L)

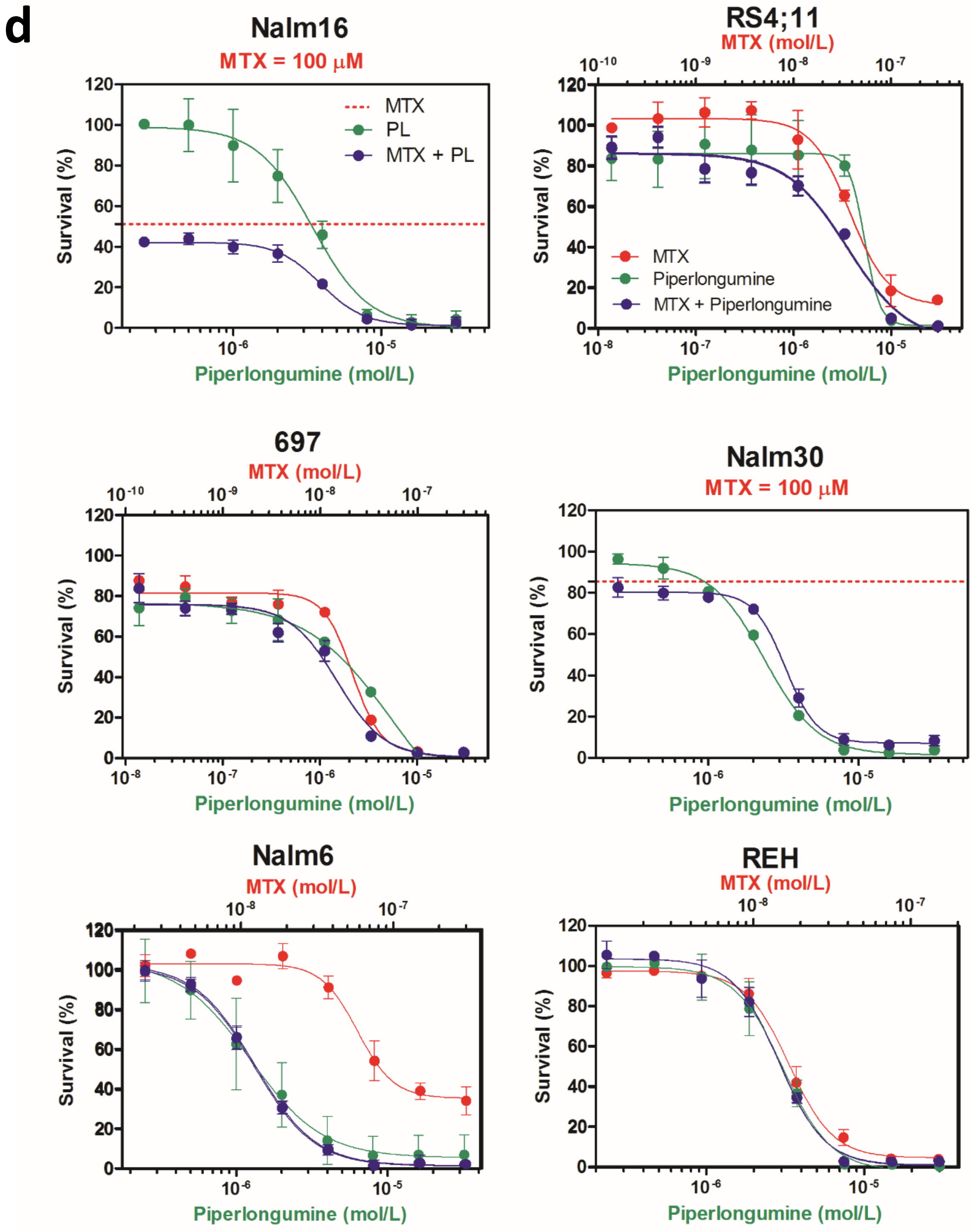
d
Nalm16
MTX = 100 μM
MTX
PL
MTX + PL
Survival (%)
Piperlongumine (mol/L)
RS4;11
MTX (mol/L)
MTX
Piperlongumine
MTX + Piperlongumine
697
Nalm30
Nalm6
REH

e

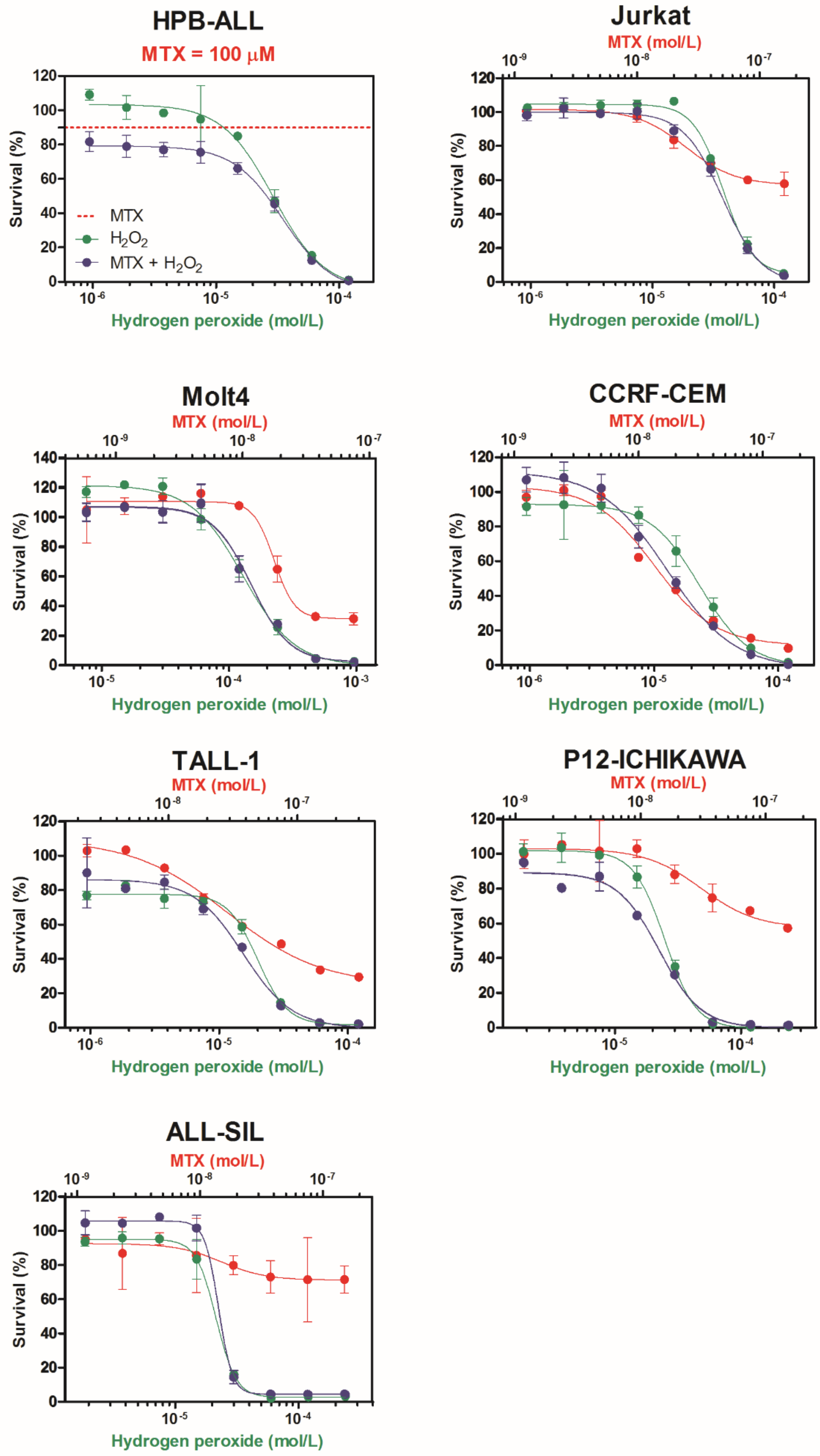

HPB-ALL
MTX = 100 µM
Survival (%)
MTX
H_2O_2
MTX + H_2O_2
Hydrogen peroxide (mol/L)
Jurkat
MTX (mol/L)
Molt4
CCRF-CEM
TALL-1
P12-ICHIKAWA
ALL-SIL

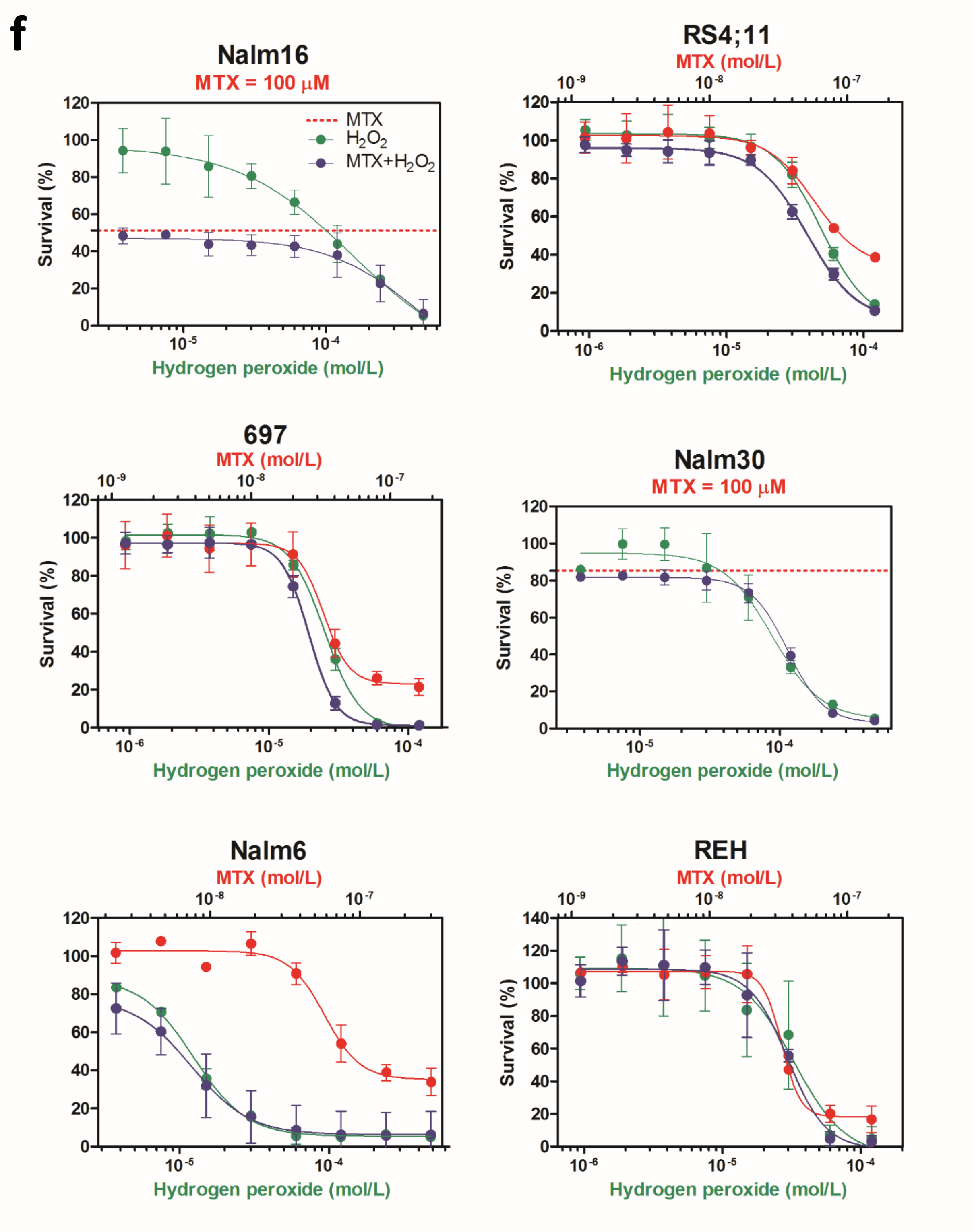

Supplementary Figure 8. Dose-response curves of **(a)** T-ALL and **(b)** BCP-ALL cell lines to MTX, in co-treatment with PEITC (3 micromol/l, green; 6 micromol/l, red) or vehicle only (blue). A.U. = arbitrary units. **(c)** T-ALL and **(d)** BCP-ALL cell lines were treated with MTX (red), piperlongumine (green) or both (blue). **(e)** and **(f)** hydrogen peroxide was used instead.

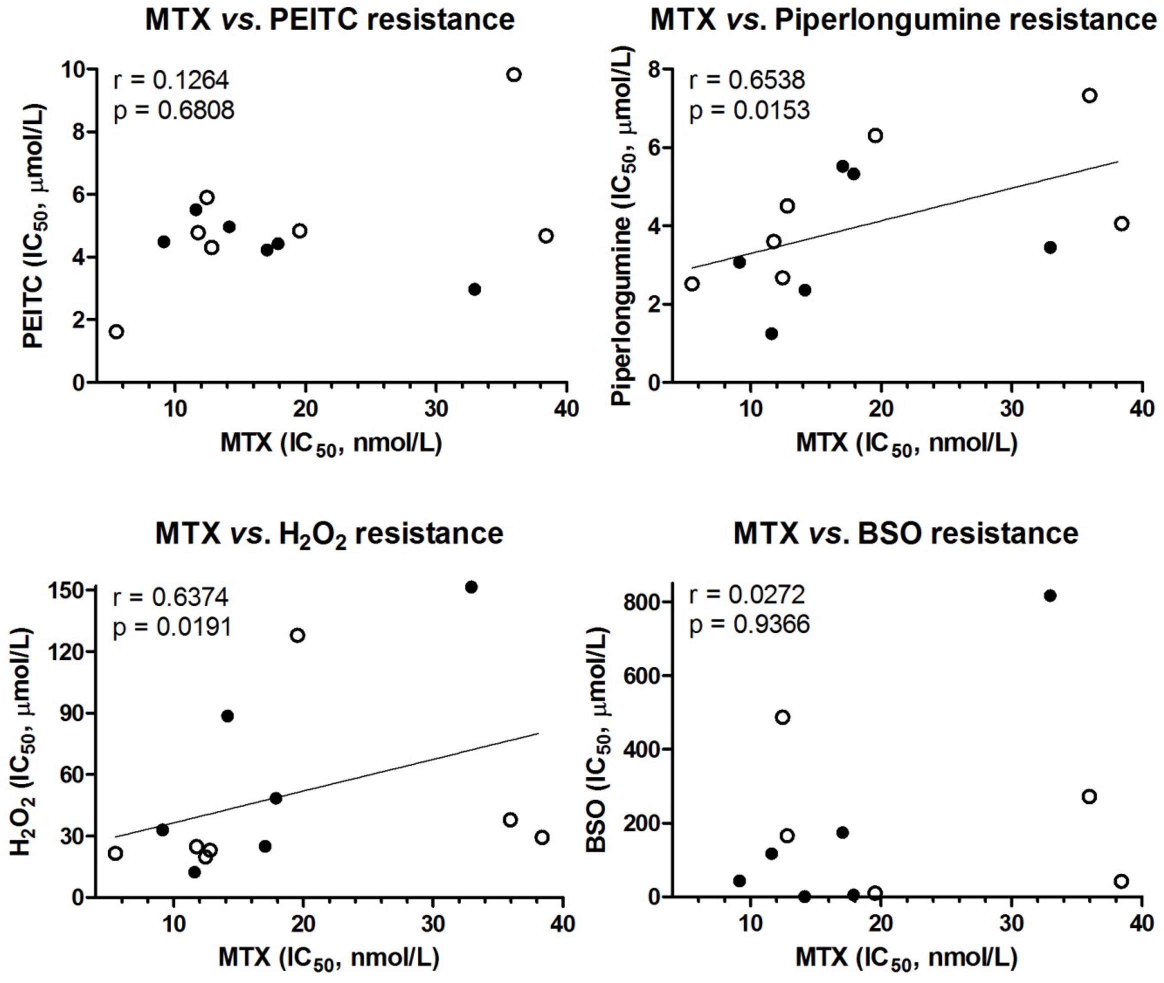

Supplementary Figure 9. Correlation between MTX, PEITC, piperlongumine, hydrogen peroxide and BSO resistance. *r* and p are from the Spearman correlation.

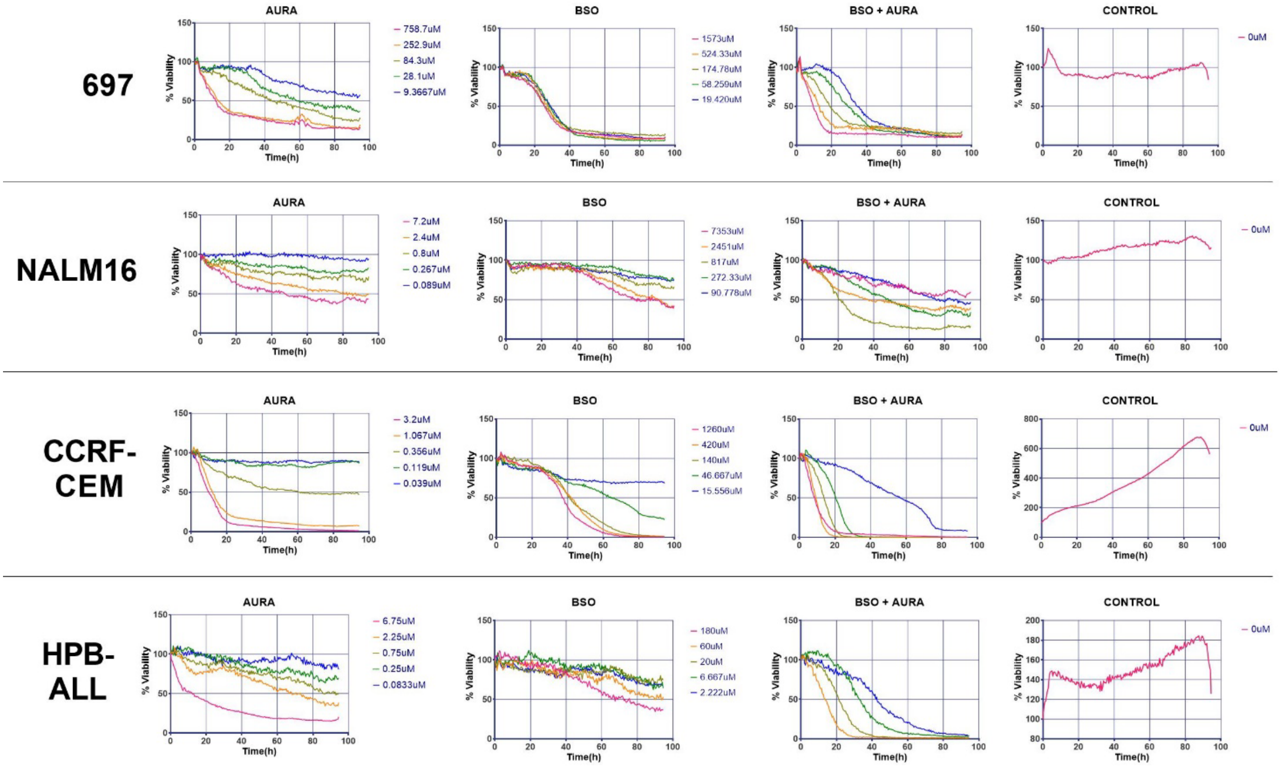

Supplementary Figure 10. Cell survival in response to Auranofin (AURA), BSO, BSO + AURA and vehicle (CONTROL) accessed by bright field imaging (high throughput drug combination screening assay) over 96 hours. In the BSO + AURA plots, curve colors indicate the corresponding doses of the single agents that were combined.

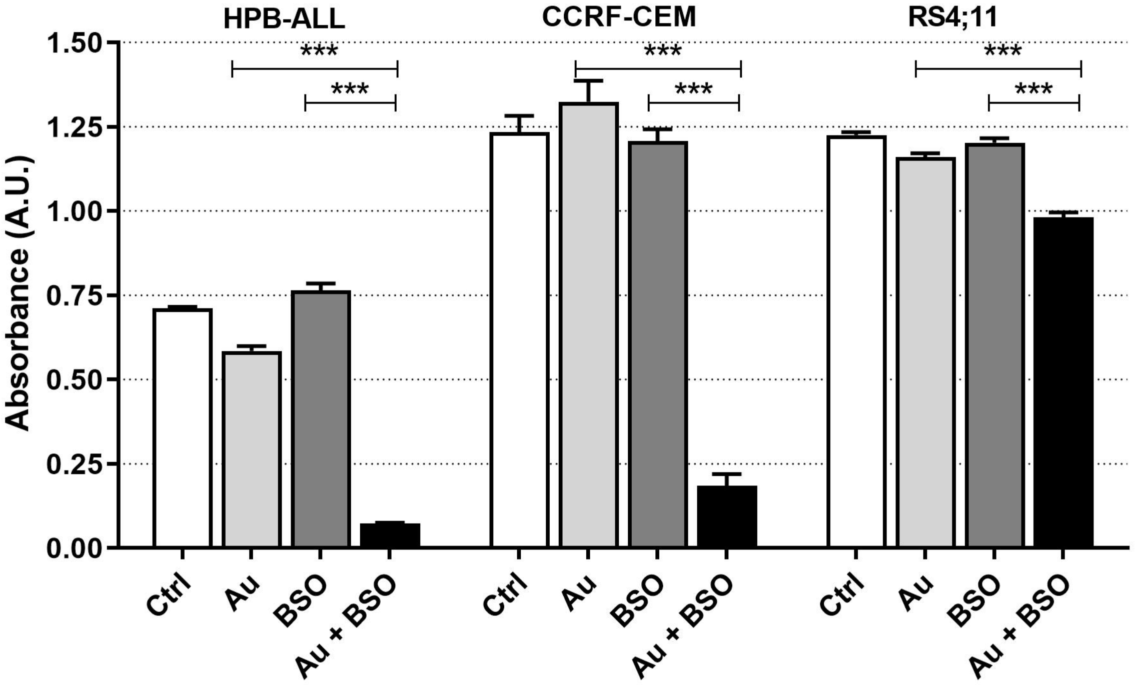

Supplementary Figure 11. Viability of cells treated with auranofin (Au), buthionine sulphoximine (BSO), and both agents combined accessed by the MTT reduction assay after 96 hours of treatment. Doses used: HPB-ALL (Au: 0.75 μM, BSO: 20 μM), CCRF-CEM (Au: 0.35 μM, BSO: 140 μM), RS4;11 (Au: 0.16 μM, BSO: 5 μM). ANOVA followed by Tukey's post-test was used to compare the histogram bars. *** = $P < 0.0001$.

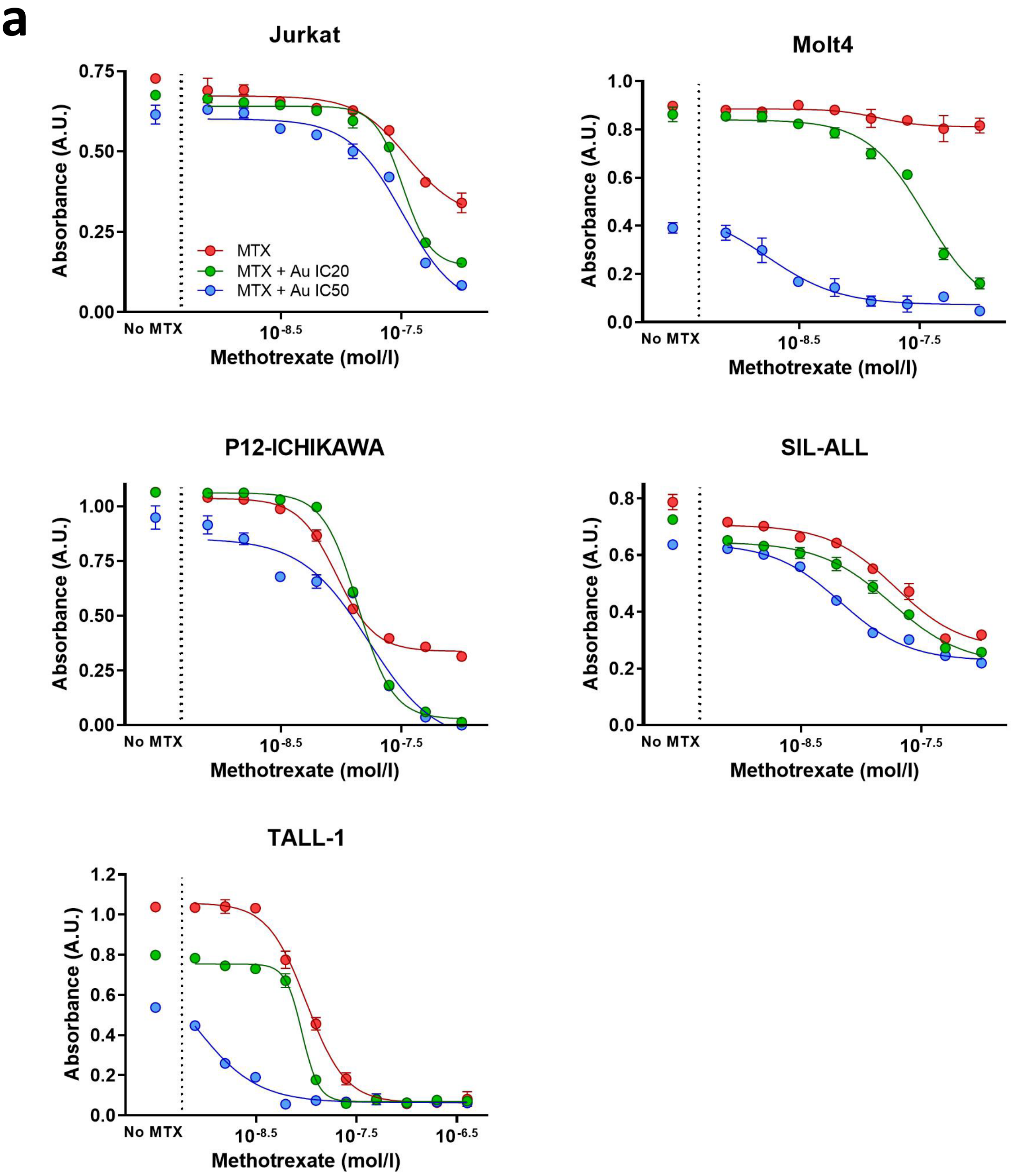
a
Jurkat
Molt4
P12-ICHIKAWA
SIL-ALL
TALL-1
Absorbance (A.U.)
Methotrexate (mol/l)
No MTX
MTX
MTX + Au IC20
MTX + Au IC50

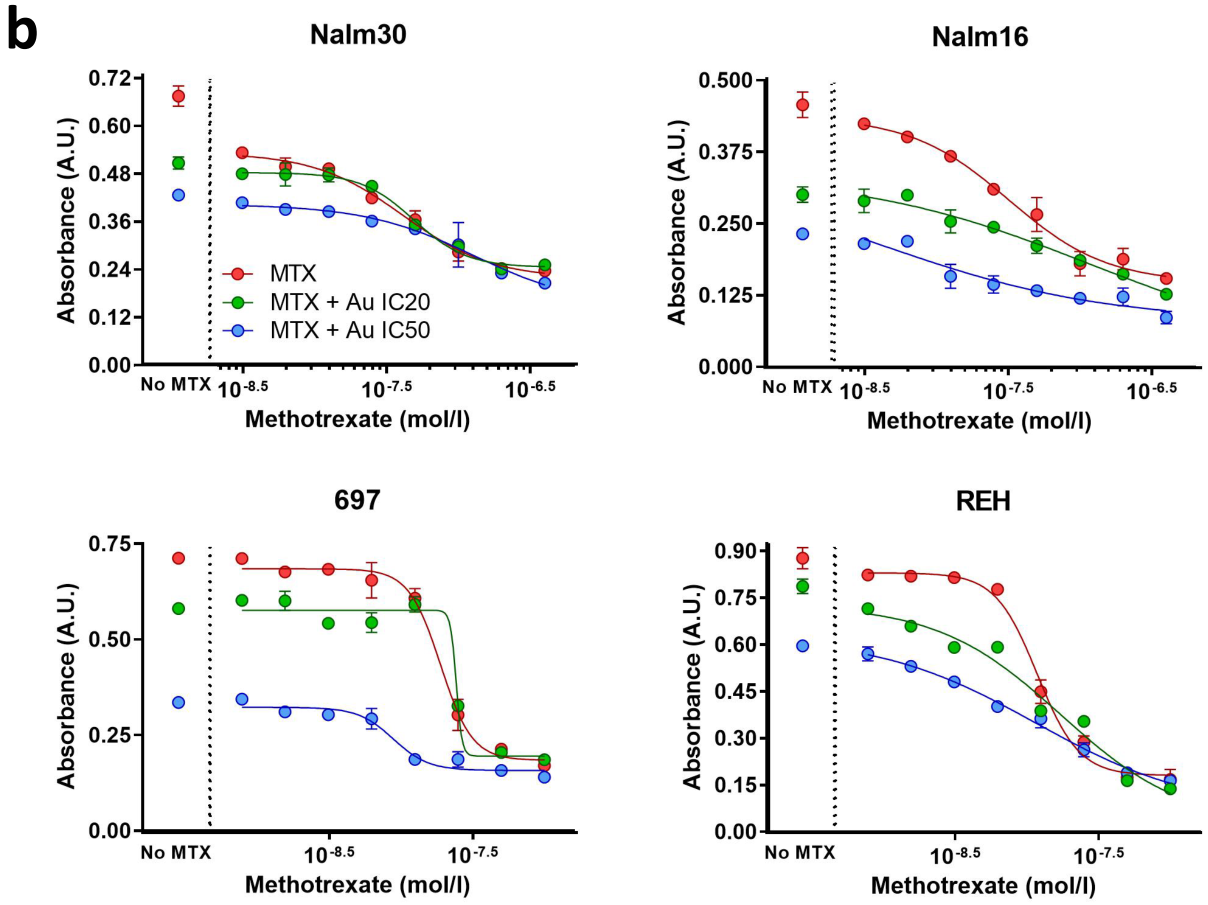

Supplementary Figure 12. Dose-response curves of **(a)** T-ALL and **(b)** BCP-ALL cell lines to MTX in co-treatment with fixed doses of auranofin for 96 hours. Doses of auranofin used: Jurkat: IC20 = 1.15 µM, IC50 = 1.68 µM; Molt4: IC20 = 1.15 µM, IC50 = 1.68 µM; P12-ICHIKAWA: IC20 = 0.7 µM, IC50 = 1.25 µM; SIL-ALL: IC20 = 50 nM, IC50 = 75 nM; TALL-1: IC20 = 150 nM, IC50 = 290 nM; Nalm30: IC20 = 200 nM, IC50 = 445 nM; Nalm16: IC20 = 0.5 µM , IC50 = 0.8 µM; 697: IC20 = 25 nM, IC50 = 85 nM; REH: IC20 = 130 nM, IC50 = 180 nM.

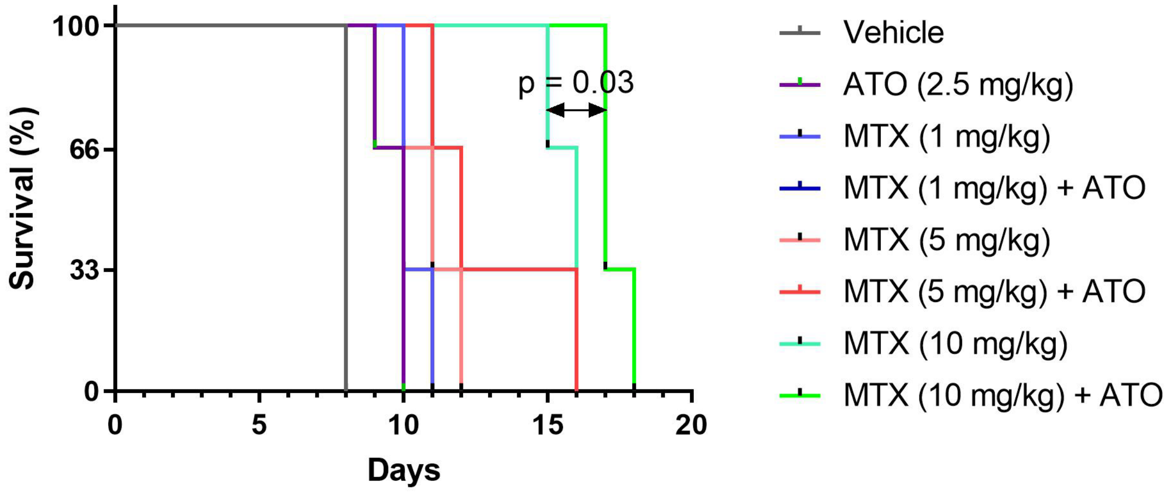

Supplementary Figure 13. Combination effect of ATO+MTX in primary B-cell precursor ALL from a murine oncogenic mutant IL7R$^{<CPT>}$ knock-in model. While MTX at 10 mg/kg improved survival over lower doses, co-treatment with ATO extended overall survival significantly. N = 3 for each treatment group, *P*-value for Log-rank test.

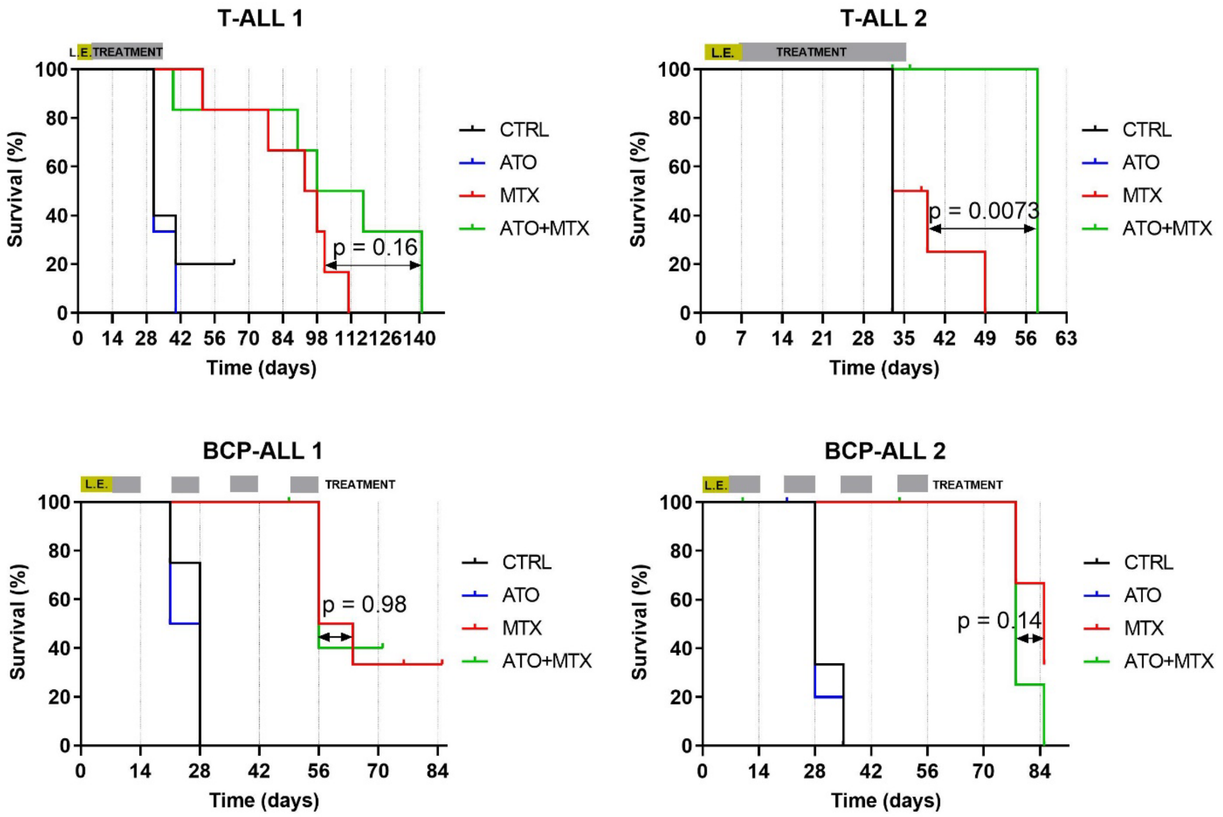

Supplementary Figure 14. Kaplan-Meier survival curves of NSG mice (6 animals per group) transplanted with 2 BCP- or 2 T-ALL primary cells. Treatment started one week after engraftment when hCD45+ cells > 0.5% in half of the mice (Day 7) and was administered for 4 consecutive weeks in T-ALL or every other week for BCP-ALL. Death and percentage of $hCD45^{+}$ cell in the peripheral blood greater than 25% were considered events (see Methods for reference). Animals that died due to treatment toxicity were censored. Doses used: MTX = 10 mg/kg daily (T-ALL) or 5 mg/kg daily (BCP-ALL); ATO = 2.5 mg/kg daily; ATO was administered 6 hours after MTX in the co-treatment cohort. L.E. = leukemia engraftment period (first 7 days post-transplantation). *P*-value for Log-rank test.

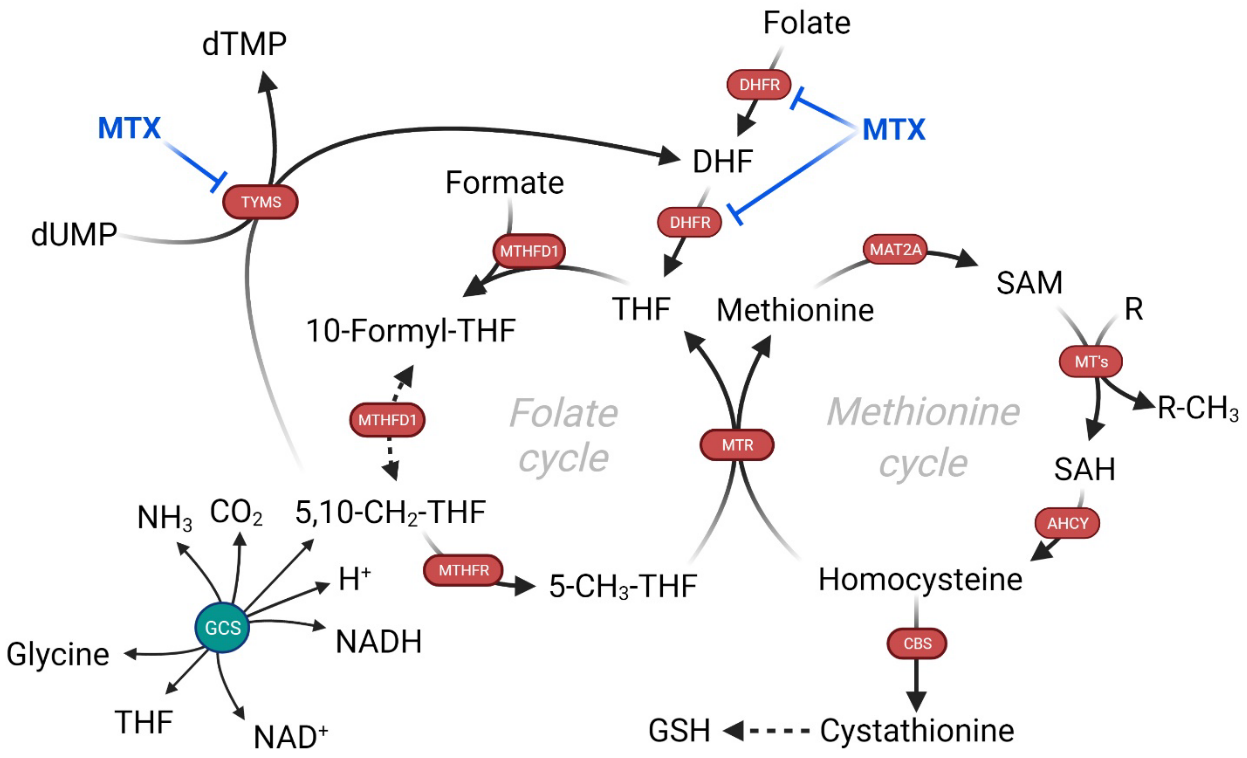

Supplementary Figure 15. The interlink between folate and methionine cycles. A scheme of the crosstalk between the folate and methionine cycles, alongside with MTX target enzymes. THF: tetrahydrofolate; 5,10-CH_2-THF: 5,10-methylenetetrahydrofolate; 5-CH_3-THF: 5-methyltetrahydrofolate SAH: S-adenosylhomocysteine; SAM: S-adenosylmethionine; R: methyl acceptor; R-CH_3: methylated product; GCS, glycine cleavage enzyme complex; MTHFD1, methylenetetrahydrofolate dehydrogenase, cyclohydrolase and formyltetrahydrofolate synthetase 1; MTHFR, methylenetetrahydrofolate reductase; MTR: 5-methyltetrahydrofolate-homocysteine methyltransferase; MAT2A: methionine adenosyltransferase 2A; MT's: methyltransferases; AHCY: adenosylhomocysteinase; CBS: cystathionine beta-synthase; TYMS: thymidylate synthetase; DHFR: dihydrofolate reductase. Created with BioRender.com.
